# Synergistic effects produced by certain antioxidants in valuable functional foods from the Romanian markets

**DOI:** 10.3389/fnut.2025.1558597

**Published:** 2025-06-19

**Authors:** Petre Savescu, Ovidiu Tita, Maria Magdalena Poenaru, Catalin Nistor

**Affiliations:** ^1^Laboratory of Bioengineering and Biotechnology, Department D31, Institute INCESA, University of Craiova, Craiova, Romania; ^2^Faculty of Agricultural Sciences Food Industry and Environmental Protection, Research Center in Biotechnology and Food Engineering, University Lucian Blaga of Sibiu, Sibiu, Romania; ^3^Laboratory of Bioengineering and Biotechnology, Institute INCESA, University of Craiova, Craiova, Romania; ^4^Department of Mining Engineering, Topo and Construction, University of Petrosani, Petrosani, Romania

**Keywords:** carrots, natural preservatives, NAD, FMN, antioxidants, antimicrobial activity

## Abstract

Today, people should change their lifestyles and nutrition to prevent metabolic diseases characteristic of our century. Functional foods, rich in antioxidants, greatly help combat everyday stress and ensure optimal nutritional density. This study is part of an applied research and experimental development program for valuable functional foods that are promoted in the Romanian market. In this research—conducted from 2020 to the present at the INCESA Institute for Applied Science Research at the University of Craiova, Romania—the occurrence and optimization of redox processes and synergistic effects in organic carrot juices were tested. These effects arise from the interaction between certain antioxidants (beta-carotene, lycopene, vitamin A) in raw organic juice and the food additives used in manufacturing recipes. Optical analysis methods (UV–Vis molecular absorption spectroscopy and atomic absorption spectroscopy) were used to monitor the technological processes, while supercritical fluid extraction with activated bio-membranes and combined field induction technology (plasmatic, magnetic, gravitational) were used to optimize certain operations and activate valuable biocomponents. The study confirmed the research hypothesis that the proposed new activation technology optimized the reaction mechanisms and synergistic and antagonistic effects occurring in the processing of organic carrots. This resulted in new functional food variants that are much richer in valuable activated bio-compounds and compositionally stable during long-term storage. Furthermore, the application of new technologies and the optimization of synergistic effects led to the development of protective mechanisms against *Escherichia coli*-type microorganisms in the final juice products. The study provides novel and valuable insights into the design and development of organic juices with optimized nutritional density (functional foods). This is extremely important for consumers and the shelf life of the product.

## 1 Introduction

The carrot belongs to the family *Apiaceae (Umbelliferae)*, within the genus *Daucus*, and has the scientific name *Daucus carota L.* The carrot is a root vegetable with multiple uses in the food industry (juices, nectars, purees, and various traditional products). The main processing route, in this case, is the juice industry.

Carrots are vegetables rich in antioxidants, vitamins, and dietary fiber while containing very few calories (only 41 Kcal per 100 grams of carrots) and little vegetable fat (1–5). In fact, just 100 grams of carrots cover the recommended daily amount of vitamin A and *β*-carotenes, and carrots are also a good source of B vitamins, vitamin K, folic acid, and potassium (6–8). For example, 100 g of fresh carrot contains 8,285 μg of beta-carotene and 16,706 I. U. of vitamin A. Beta-carotene is one of the most powerful natural antioxidants, protecting the body against harmful free radicals (9–16). In the liver, carotenoids are transformed into vitamin A, which is essential for visual acuity, reproduction, maintenance of epithelial (skin) integrity, growth, and development (17–19). Fresh carrots are also rich in vitamin C (with 5.9 mg of vitamin C in 100 grams of carrots), representing almost 10% of the Recommended Dietary Allowance (RDA) (20–25). The role of vitamin C in the body is very well known (26–30). It acts as both an antioxidant and a protector for healthy connective tissue and in many metabolic processes (31). Fresh carrots contain significant amounts of B vitamins: folic acid, vitamin B6 (pyridoxine; 100 g represents 10% of the RDA), vitamin B1 (thiamine; 6% of the RDA), vitamin B5 (pantothenic acid; 5.5% of the RDA), vitamin B3 (niacin; 6% of the RDA), vitamin B2 (riboflavin; 4% of the RDA), and fiber (32–39). All of these are bioactive compounds that work as enzyme co-factors in catalyzing biochemical processes in the body (2). The relevant published literature indicates that flavonoids in carrots act as protective factors against oral cavity, lung, and skin cancers (40, 41). Vitamin K, which is involved in certain redox processes beneficial for the body, is also present in carrots, with 13.2 μg of vitamin K in 100 g of carrots (almost 11% of the RDA) (42–44). In addition to these vitamins, carrots also contain important amounts of minerals: potassium, manganese, phosphorus, calcium, and copper (45). One hundred grams of carrots contain 320 mg of potassium (6.5% of the RDA) (46, 47). This mineral is important for maintaining a good heart rate and blood pressure, as well as in minimizing the effects of sodium in food (48). Manganese is present in an amount of 0.143 mg/100 g of carrots (7.2% of the RDA) (49). This element acts as a co-factor for a very important redox enzyme (superoxide dismutase, SOD) (50).

The foods of the future will be based on organic raw materials and will be energized through activation, both during the production of agricultural raw materials and during processing (51–56). They will be characterized by medium and small volumes but high specific energy (due to their high nutrient density). In addition, all new technologies must be eco-friendly to ensure sustainable development (57). The use of mixed-field technologies (plasmatic, magnetic, and gravitational) will induce a high natural energy charge, suitable for long shelf-life due to certain bio-compounds with antioxidant value (58–60). These will oxidize first, thereby protecting the environment they come from (61–64). This can help maintain a long natural shelf life for products, simply by monitoring the activity of some NADH+H^+^- or FMNH+H^+^-dependent oxidoreductases (65–67).

In Romania, there are more than 1,500 different types of medicinal, aromatic, and spicy plants that can be used as sources of raw materials for the development of functional foods or valuable food supplements. Fruits from Romania (berries, fruits, and vegetables) have a high antioxidant potential, showing increased concentrations of anthocyanins, polyphenols, vitamins, and provitamins (68). This antioxidant potential and the “Oxygen Radical Absorbance Capacity (ORAC)” scale in fruit are the best measures in the fight against cancer and other diseases considered incurable. To avoid malignant risks, specialists have established that the optimum daily requirement of plant substances capable of absorbing free oxygen radicals should exceed 5,000 ORAC units (69).

One of the main specific disadvantages of these antioxidants is the decrease over time of this ORAC figure in recent years (70). Another problem is related to preserving the antioxidant character after the processing of the raw materials (this is why it is recommended to use mild food processing techniques such as supercritical fluid extraction, SFE) (59, 68). An important problem that needs to be resolved quickly is how these raw materials (rich in antioxidant compounds) should be preserved so that this antioxidant character can be maintained for a long time.

This article proposes improvements to working methods by developing a reagent-free method that minimizes disturbing factors, while maintaining low analysis costs and meeting all the requirements for precision, repeatability, reproducibility, and accuracy specific to top scientific research. The article also presents an analysis model for a type of vegetable (Romanian carrots) rich in provitamins A (alpha- and beta-carotenes, lycopene) and vitamin A, which have extremely useful antioxidant properties but are paradoxically underutilized.

To effectively use these plant resources in the design and construction of valuable food supplements (antioxidant-rich nutraceuticals), it is crucial that the raw materials used (fruits, leaves, flowers, herbs, roots) are clean. This means they must be free of growth or ripening hormones, residues from phytosanitary treatments, pesticides, and heavy metals. All of this is necessary because organic raw materials are prohibited for use according to the requirements of Reg. (EU) 848/2018 in its updated/consolidated form, and their antioxidant capacity must be monitored during processing and storage. Additionally, the raw materials used must be free from pathogenic flora or attacks by pathogenic microorganisms, which could cause DNA changes in the environment and health problems for consumers.

This work is part of a complex project in which the behavior of the main oxidoreductase enzymes (NAD- and FMN-dependent) acting in certain antioxidant-rich plants has been analyzed. Through multiple physical–chemical–microbiological tests and laboratory analyses, it has been demonstrated how the ratio of the concentrations of oxidized and reduced forms of these co-enzymes can serve as markers for analyzing antioxidant capacity—both at the liquid (solid)/air interface (where FAD- or FMN-dependent enzymes predominantly act) and within food supplements (where NAD-dependent enzymes predominantly act after activation). For the sake of simplicity, only the experimental aspect was selected, focusing on improving the extraction yield of certain bio-compounds with antioxidant potential from Romanian carrots (Bogdan Variety).

The antioxidants obtained by extraction from this type of carrot are based on type A provitamins (alpha- and beta-carotene, lycopene) and type A vitamins. In addition, the activity of some NADH+H^+^- or FMNH+H^+^-dependent oxidoreductases in carrots can be considerably enhanced in the processing environment. Driving redox processes controlled by these oxidoreductases to areas of low redox potential also creates a strong antimicrobial and anti-helminthic effect (against the carrot root nematode *Meloidogyne hapla*, pests of carrots grown in organic systems) (10, 59). The article also highlights the kinetics of the concentration ratios of oxidized and reduced forms (an important and variable part of the Nernst equation for oxidation–reduction potential). Thus, it is emphasized how the result of a complex field is manifested—in preserving the antioxidant capacity, restoring it, and increasing the duration of action of food supplements using this innovative technique.

In November 2024, the Federal Centers for Disease Control and Prevention in the USA announced that an outbreak of *E. coli* has infected dozens of people who ate bagged organic carrots, resulting in one death. Altogether, 39 people were infected, and 15 were hospitalized in 18 states after eating organic whole and baby carrots (71).

According to relevant EU regulations, the maximum standards for microorganisms in carrots are …. (54):

- Yeasts and molds, max, c.f.u./g: 500
- *Escherichia coli*, max, c.f.u./g: 10; *Enterobacteriaceae*, max, c.f.u./g: 10
- Coagulase-positive *Staphylococcus*, max, c.f.u./g: 10
- *Salmonella,* c.f.u./25 g: absent

When operations that enhance antimicrobial activity are integrated into processing technology, food safety and consumer health increase.

## 2 Materials and methods

### 2.1 Methods used in analysis of raw materials

The moisture—according to SR ISO 712: 2010; the acidity—according to SR 90: 2007 and SR ISO 750: 2008; total Soxhlet fats—according to EC Regulation 152/2009; the nitrogen content (proteins)—according to SR EN ISO 20483: 2014; total ash—according to SR ISO 2171: 2010; the fiber content—according to AOAC 991.43/1995; the nitrite content of water from hydroalcoholic extract—according to SR EN 26777: 2002/C91: 2006; the nitrate content of water from hydroalcoholic extract—according to APHA (American Public Health Association); water pH—according to SR EN ISO 10523: 2012; the mineral substances—using atomic absorption spectrometry (AAS); analysis of the total number of aerobic mesophilic germs (NTG)—according to SR EN ISO 4833-1: 2014 and SR EN ISO 4833-2: 2014; the number of mold cells—according to SR ISO 21527-1: 2009; identification of DNA changes due to the attack of pathogenic microorganisms—with the help of the mathematical coprocessor from the UV–Vis T92 plus Spectrometer; detection and counting of colony-forming microorganisms—according to SR EN ISO 4833-1: 2014; the mercury ion content—using cold vapor generating atomic absorption spectrometry (CVAAS)—according to SR EN 13805:2015; the lead and cadmium content—using flame atomic absorption spectrometry (SAAFL)—according to SR EN 13804: 2013; the total arsenic content—using hydrogen generator atomic absorption spectrometry (HGAAS) according to SR EN 13804: 2013 (72).

### 2.2 Use of the UV–Vis molecular spectroscopy in the management of food additives in carrot juice

#### 2.2.1 Experimental variants preparation

For the main experimental variant, a Romanian type of carrot (the Bogdan variety) was used. The carrots were washed, peeled, cut into rounds, and pressed to obtain the basic juice. This primary carrot juice, without additives, is rich in vitamin A precursors (alpha and beta-carotenes, lycopene). This juice was enhanced with the extract obtained by Supercritical Fluid Extraction (SFE) (in a ratio of 1:20) and thus constituted the V0 Witness Variant.

By using permitted food additives, the experimental variants V_1_-V_10_ were realized: V_0_—variant of non-additive carrot juice (Witness Variant 1);

- V_1_—variant of carrot juice (V_0_) with preservative (acetic acid, 1%);
- V_2_—variant of carrot juice (V_0_) with preservative (salicylic acid, 1%);
- V_3_—variant of carrot juice (V_0_) with carmine dye (E120, 1%);
- V_4_—variant of carrot juice (V_0_) with *β*-carotene dye (E 160a, 1%);
- V_5_—variant of carrot juice (V_0_) with a natural sweetener (sugar 3 g/100 mL);
- V_6_—variant of carrot juice (V_0_) with a natural sweetener (stevioside 3 g/100 mL);
- V_7_—variant of carrot juice (V_0_) with a synthetic sweetener (fructose 4%);
- V_8_—variant of carrot juice (V_0_) with sugar (3 g/100 mL) and carmine dye (1%);
- V_9_—variant of carrot juice (V_0_) with carmine dye (1%) and stevioside (3 g/100 mL);
- V_10_—variant of carrot juice (V_0_) with additives, according to a commercial juice recipe.

From the carrot residues left after pressing, another juice is obtained through diffusion and filtration. This juice is then activated in the plasma field—with the help of special generators—as shown in the specific figure, becoming the activated V0 variant (V0A).

This juice contains the same lines of food additives as in the case of the V0 variant.

On the same principles, the following additive variants are obtained from the V0A variant (activated juice):

- V_0A_—variant of carrot juice without additives (Witness Variant 2);
- V_1A_—variant of juice (V_0_A) with preservative (acetic acid, 1%);
- V_2A_—variant of juice (V_0_A) with preservative (salicylic acid, 1%);
- V_3A_—variant of juice (V_0_A) with carmine dye (E120, 1%);
- V_4A_—variant of juice (V_0_A) with a β-carotene pigment (E 160a, 1%);
- V_5A_—variant of juice (V_0_A) with a natural sweetener (sugar 3 g/100 mL);
- V_6A_—variant of juice (V_0_A) with a natural sweetener (stevioside 3 g/100 mL);
- V_7A_—variant of juice (V_0_A) with a synthetic sweetener (fructose 4%);
- V_8A_—variant of juice (V_0_A) with sugar (3 g/100 mL) and carmine dye (1%);
- V_9A_—variant of juice (V_0_A) with carmine dye (1%) and stevioside (3 g/100 mL);
- V_10A_—variant of juice (V_0_A) with additives, according to a commercial juice recipe.

At this stage, the changes produced by certain food additives on bioactive compounds with antioxidant capacity— which are included in the composition of valuable food supplements—were also highlighted.

The analysis of these experimental variants allows the development of the best management of food additives in order to create the best food supplement recipes. In the final recipes, only the sensorily improved experimental variants will be included, provided that the antioxidant capacity is not affected.

Molecular absorption spectra curves were generated using a UV–Vis spectrophotometer type T92 Plus, manufactured by PG Instruments, U. K. The spectrophotometer was set to operate at a 1 cm bandwidth and to record nanometer-by-nanometer molecular absorption values in both the UV (190–400 nm) and visible (400–700 nm) ranges. Thus, the curves of molecular absorption spectra were obtained for each experimental variant (series V0-V10, and V0A-V10A) in the two wavelength ranges, UV and VIS.

The equipment automatically recorded the spectral curves while switching between deuterium and tungsten lamps at 362 nm via automatic programming. To verify the obtained values, at each measurement, the T92 Plus spectrophotometer was set to develop an automatic retracking.

To measure molecular absorption, special parallelepiped UV quartz cells with a square side in a section of 1 cm were used.

#### 2.2.2 UV–Vis spectroscopy

It is an optical technique that utilizes calibration curves and the *“Single Addition Method”*—with pure analytical substances (from Merck GmbH) used in constructing concentration scales—in order to determine the peaks of the molecular absorption spectra of the main bio-compounds in solution. In addition, calibrations were conducted using reference media (RM) or certified reference media (CRM). All this was done to comply with the quality and traceability policies—rigorous requirements in the practice of physical, chemical, and microbiological testing laboratories certified to the EN ISO/IEC 17025:2018 Standard (File 1,” Preparing Samples for Analysis—Supplementary material 2, details”).

All samples were analyzed in duplicate; the quantification of the components of the expanded uncertainty was achieved by calculating the measurement uncertainty while considering the influence exerted by all the equipment involved in the determination.

The UV-WIN software runs on various Windows operating systems, providing full control over the instrument and accessories, as well as data collection and processing.

By determining the wavelength at which the molecular absorption maxima are recorded, it was possible to ascertain the concentrations of nicotinamide adenine (NAD, oxidized form), nicotinamide adenine dinucleotide (NADH+H^+^, reduced form), flavin mononucleotide (FMN, oxidized form), flavin mononucleotide (FMNH+H^+^, reduced form), and the kinetics of some valuable bio-compounds over time. The ratios of the concentrations of the reduced and oxidized forms of these important enzyme co-factors provided the best picture of the redox potential variation both within the solution and at the liquid/air interface. The redox potential is dependent on the logarithm of the concentrations of the oxidized and reduced forms of NAD or FMN (according to the Nernst equation—Supplementary Figure 1A). The analysis of the variation of these concentrations over time provided a clear image of the retention time of their antioxidant activity. All of this is extremely important for analyzing the antioxidant activity and antioxidant capacity of the vast majority of antioxidant agents in food.

### 2.3 Determination of DNA changes induced by the attack of pathogenic microorganisms

Very important is the use of healthy raw materials, free from heavy metals, pesticide traces, plant hormone traces, and signs of attack by pathogenic microorganisms. Measurement of DNA changes, long before the attack of pathogenic microorganisms under the microscope, was performed using the DNA/Proteid Detector v.5.0 with the DNA/Proteid Biology Chipset DP 200307, which allows for automatic measurement of the absorbance ratio A1 at 260 nm and A2 at 230 nm (presented in Supplementary Figure 6). The related equipment and software are attached to the T92 plus UV–Vis spectrometer, produced by PG Instruments, UK, which measures the absorbances of various bio-compounds in the UV range (190–400 nm) and visible range (400–700 nm). For accurate determination of concentrations (by analyzing the peaks of the molecular absorption spectra) of bio-compounds, the single addition method and calibration curves were used. This equipment uses an Optical System with a Czerny-Turner Monochromator featuring high-resolution grating, wavelength reproducibility of 0.1 nm, wavelength accuracy of ± 0.3 nm (automatic wavelength correction), photometric accuracy of ± 0.004 Abs (0.5–1.0 A), photometric reproducibility of 0.001Abs (0–0.5 A), baseline flatness of ± 0.001 Abs, baseline stability of 0.0004 Abs/Hr (500 nm, after preheating), and photometric noise of ± 0.001Abs (500 nm, 0 Abs, 2 nm spectral bandwidth). Using bio-membranes in the cold plasma field influences the attacked samples and can increase the precision of measurements in this case (Supplementary Figure 7) (59).

### 2.4 Atomic absorption spectroscopy (AAS)

In the first phase, sample preparation for atomic absorption spectrometry involved mineralization (microwave digestion). Microwave digestion is used to prepare samples of all types (plant, soil, food, pharmaceuticals) for elemental analysis by inductively coupled plasma (ICP), inductively coupled plasma mass spectrometry (ICP-MS), or atomic absorption (AA), which require the sample to be in solution form for introduction into the analyzer. Acid digestion is employed to break down the sample matrix, leaving the elements of interest in solution and ready for analysis. CEM microwave digestion systems rapidly break down a wide variety of sample matrices, leaving behind a clear solution containing the analytes of interest (26).

For this purpose, a CEM Mars system of microwave mineralization, 1,200 W, was used. The MARS system from CEM is a multi-mode platform equipped with a magnetic stirring plate and a rotor that allows for the parallel processing of several vessels per batch. We used the HP-500 (Teflon (TFA) insert) (vessel volume 80 mL, max pressure 350 psi, max temperature 210^o^ C) and Greenchem (glass borosilicate insert) (vessel volume 80 mL, max pressure 200 psi, max temperature 200^o^ C) vessel assemblies, both based on a 14-position rotor. The system delivers a continuous power output between 0 and 1,200 W. Temperature was controlled internally by a fiber optic probe in one control reference vessel.

*Method:* In summary, the substance was weighed to an analytical precision of 10.00 g of dry substance (d.s.). For the mineralization step, 10.00 g of the product (carrots in their raw state) were added to each digestion cartridge, along with 6 mL of concentrated nitric acid and 3 mL of 30% hydrogen peroxide. To create a blank, one digestion cartridge was used without the addition of the product, but with the reagents (59, 68). This method is outlined in Table 1.

**Table 1:** The microwave digestion steps (in the context of A. A. S. Procedure).

Power, W	Time, min	Agitation	Comment
300	4	Yes	To protect the cartridges
0	2	No	To assist in the sedimentation process in the cartridge
400	4	Yes	
600	3	Yes	
200	1	No	
800	3	Yes	
1,000	4	Yes	
0	10	No	Cooling to open the cartridges

For the AAS method, a Varian SpectrAA 220Z Atomic Absorption Spectrometer Furnace System was used, along with a Varian SpectrAA 220Z Auto Sampler, Varian GTA 110Z Furnace, Varian UltrAA, and associated Windows interface software.

### 2.5 Use the plasmatic field in activated the bio-compounds from carrots

An innovative cold plasma technology was used (together with specific enzymatic activators) in the concentration separation of valuable bio-compounds from carrots. As activators, acetyl-CoA (an obligatory activator of NADH-reductase activity) was used, and the rate of activity was monitored by the ratio of NAD/NADH+H^+^. In conventional research, cold plasma results from the use of a Tesla coil string. Cold plasma is produced together with static electricity, which can also release hot plasma (which can negatively affect less thermally stable biological compounds). Therefore, a novel Tesla T-wire Tesla coil string system (“T-coil in T-coil”—the inner coil is directly powered, the outer coil has a reversed power circuit, and a nano-rated CO2 gas circulates between them) was used. This innovative system eliminates any static electricity and heating from the mixture, resulting in the separation and activation of biological compounds in a stable plasma field. This technology has been used both to activate bio-membranes (to maintain a product at a certain level of plasma induction) and to improve the yield of the supercritical extraction technique—in a *Helix Natural Product* Extractor (59, 68).

Before the SFE and after diffusion, certain interfering substances were separated, as these could be drawn into the solvent or the final (aqueous) extracts. To this end, a combination of calcification and carbonation (defecation and carbonation) purifying techniques was used, as well as filtration separation techniques including special membranes. Thus, a series of soluble pectin, lignin, celluloses, and hemicelluloses were separated. The natural protective membranes are obtained from vegetal cellulose, which were pressed and charged in ionic, magnetic, and gravitational fields. Thus, active nanostructures are produced, which directly influence the activity of the co-enzymes and greatly increase the efficiency of separating the active bio-compounds. Another very important aspect is the “cold” separation of certain valuable bio-compounds in carrots under the influence of cold, which a certain field (plasma, magnetic, and gravitational) resultant force has on the valuable compounds in the hydroalcoholic extracts made from carrot pomaces.

In these extractions, Helix Natural Product SFE equipment was used, delivered to the Research Infrastructure of the Bioengineering and Biotechnologies Laboratories, Research HUB INCESA, University of Craiova, Romania—by the world leader in this field, Applied Separations, USA (Supplementary Figure 2).

This system has been improved by the use of bio-membranes loaded in the plasma field and placed at specific locations in the supercritical extraction unit. This creates a plasma field generated by the difference between the field created by the CO2 used as the supercritical extraction agent (at a temperature of 31–32°C and a pressure of 150–650 bar, depending on the bio-compound we want to separate) and the shielding field maintained by the activated bio-membranes. The use of such a system increased the extraction yield (especially between 180–350 bar) by more than 20% (59, 68).

Different compounds were extracted at different pressures. To increase the extraction yield, bio-membranes pre-charged in a plasm-inductive carbon dioxide field, amplified by nano-coupled metal electrodes (Cu, Zn, and Fe), were applied inside the central unit. This altered the pressure range for some carotenoids (optimal separations being found between 300 and 450 bar at 31–32°C) and increased the extraction yield to 120% (compared to conventional methods). An important balance was thus achieved between the internal environment (constituted by the supercritical carbon dioxide agent) and the external environment (generated by bio-membranes charged with carbon dioxide in inductive, electromagnetic, and gravitational fields).

The extract was then added to the baseline carrot juice variant, resulting in the best non-additive baseline variant, Variant V0. The use of the SFE technique produced a carrot juice that is much more active and healthier for consumers. Even though it is a “Premium” food product, production costs are expected to decrease as it scales up to semi-industrial and industrial levels.

Inductive, magnetic, and gravitational plasma field generators were used to charge the bio-membranes positioned inside the SFE extraction unit and to increase the supercritical extraction yield (Supplementary Figure 2). For bio-membrane loading, special wands were employed that utilize direct and indirect Tesla coils, with the Tesla effect being enhanced by the Garnier effect (due to the four right angles of the nano-coated Cu cylinders containing the Tesla coils, as shown in Supplementary Figure 3).

The set of generators is constructed using special assemblies made of copper cylinders, within which Tesla coils—some with a direct effect and others with a reversed effect—serve as the electromagnetic core. These coils are energized by small spheres made of anisotropic materials impregnated with solutions of Fe, Cu, and Zn metals activated at the electrodes in specialized electrolytic systems. This amplifies the combined field effect and creates an environment that prevents cell oxidation. A model of such a generator (currently in an advanced stage of patenting) is shown in Supplementary Figure 5. The combined field loading (plasma-induced, magnetic, and gravitational) of the juices and the activation of these juices (for the best additive variants) are demonstrated using a FLIR E86 thermal imaging camera and a laboratory microscope.

The effect of these innovative generators is also evident in the reduced rate of transformation from the reduced forms to the oxidized forms of the oxidoreductases present and their protection (as shown in the videos and images). The specific frequencies at which they operate are protected by international copyright laws.

The generator variants have been submitted to the Romanian authorities for further verification and authorization (to identify any potential issues regarding the food safety of the products in which they are used—through non-invasive mild food processing techniques).

### 2.6 Use the plasmatic field to protect food against pathogenic microbial attack in carrots

The mixed field is generated directly by the combined field generators (plasma, magnetic, and gravitational). It starts from the first feed to a mini-electrolysis circuit that produces (depending on the electrolyte in the bath and the voltage at the electrode terminals) a nanostructured gas that liquefies at normal laboratory temperature. This fluid is placed at the end of the direct and indirect Tesla coils, in a nano-coupled copper tube that exhibits several right angles (to cumulatively utilize the Garnier Effect). The systems (presented in Supplementary Figures 3–5) generate and maintain a mixed field that greatly aids in the preservation of agricultural and food products without the use of preservatives.

## 3 Results and discussion

### 3.1 UV–Vis molecular spectroscopy

Using Certified Reference Materials or Reference Materials and the Single Addition Method, the construction of calibration curves is straightforward.

With the help of calibration curves (constructed before the laboratory tests), the concentrations of oxidized and reduced forms of the main antioxidants in the normal experimental variants (V_1_-V_10_) and activated (V_0A_-V_10A_) could be easily determined.

**Figure 1:**
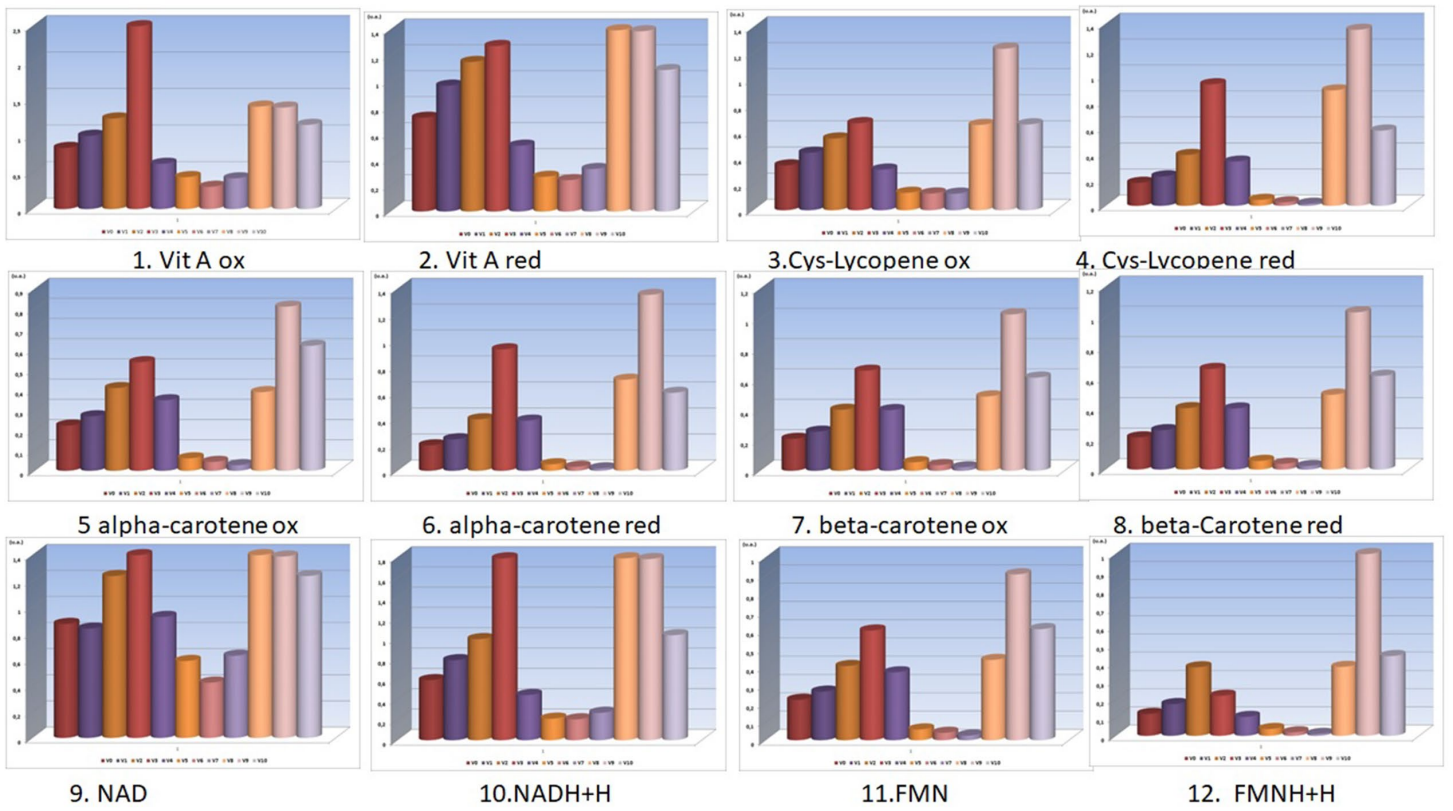
Bio-compounds molecular absorbance units (units absorbance) for V_0_-V_10_ experimental variants (the selected bio-compounds were vitamins A and provitamins A in their reduced and oxidized forms) (Supplementary Figures 1–6).

**Figure 2:**
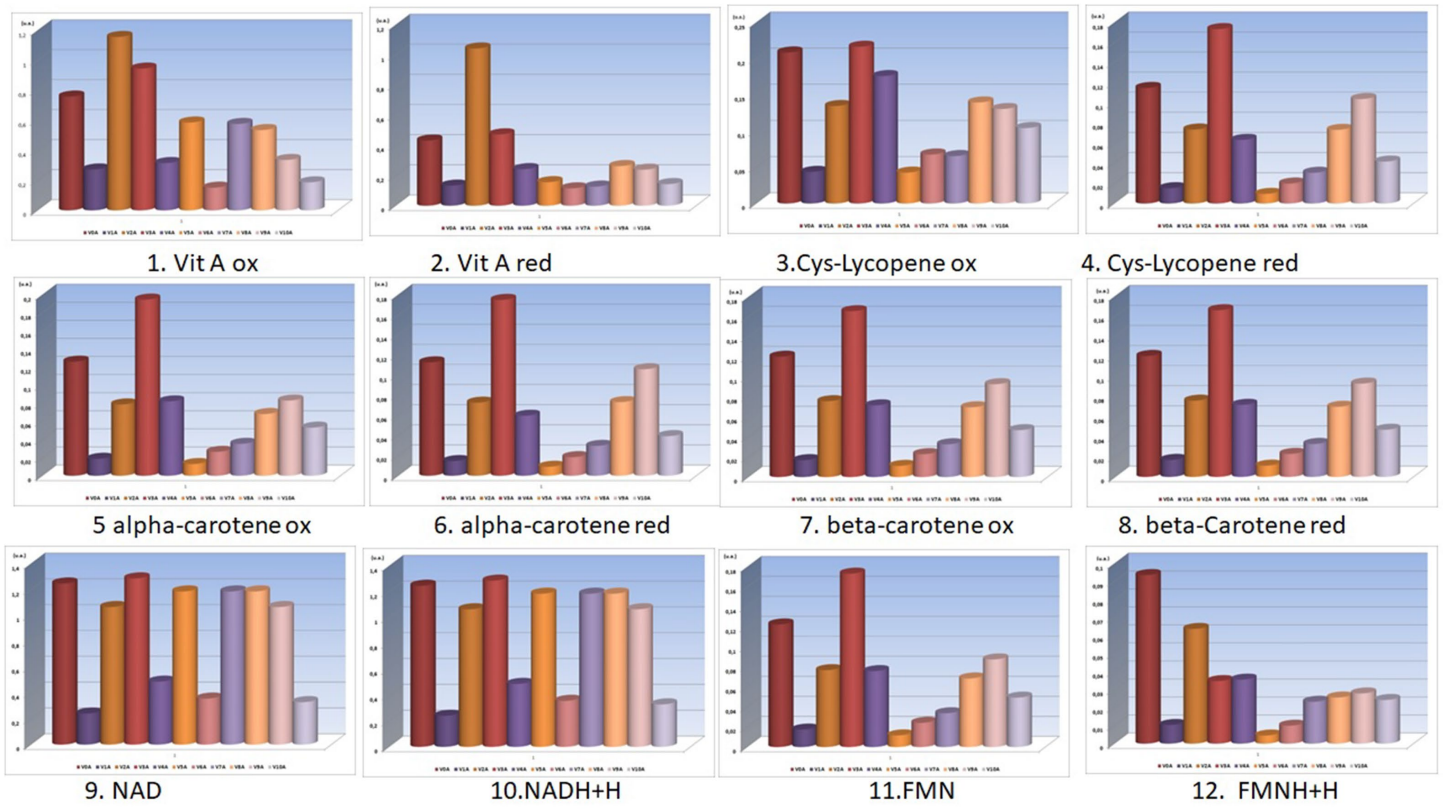
Bio-compounds molecular absorbance units (units absorbance) for V_0A_-V_10A_ experimental variants (the selected bio-compounds were vitamins A and provitamins A in their reduced and oxidized forms).

Thus, the results presented in Figures 1, 2 were obtained.

Additionally, through this optical analysis method, the molecular absorption spectra curves were obtained, which showed different trend lines and varying R^2^ statistical determination coefficients.

The nutritional density of these functional foods (the final carrot juices) obtained in these innovative processing schemes is also determined by the concentrations of the active (reduced and oxidized) forms of vitamins A and provitamins A (alpha-carotenes, beta-carotenes, lycopene), as shown in the graphs in Figures 1, 2.

In carrot juice, the activity of some compounds involved in redox processes is very important (65). The main pairs of compounds that appear are:

- NAD and NADH2 (very important for anaerobic oxidoreductase enzymes in the medium of carrot juice);
- FMN and FMNH_2_ (specific to aerobic oxidoreductases that influence oxidation or reduction at the liquid-juice/air interface and the shelf life of products);
- Reduced and oxidized hemoproteins;
- Natural pigments*—*provitamin A (reduced and oxidized lycopene; oxidized and reduced alpha-carotene forms; oxidized and reduced beta-carotene forms);
- Vitamins of type A (retinol)—oxidized and reduced forms.

**Figure 3:**
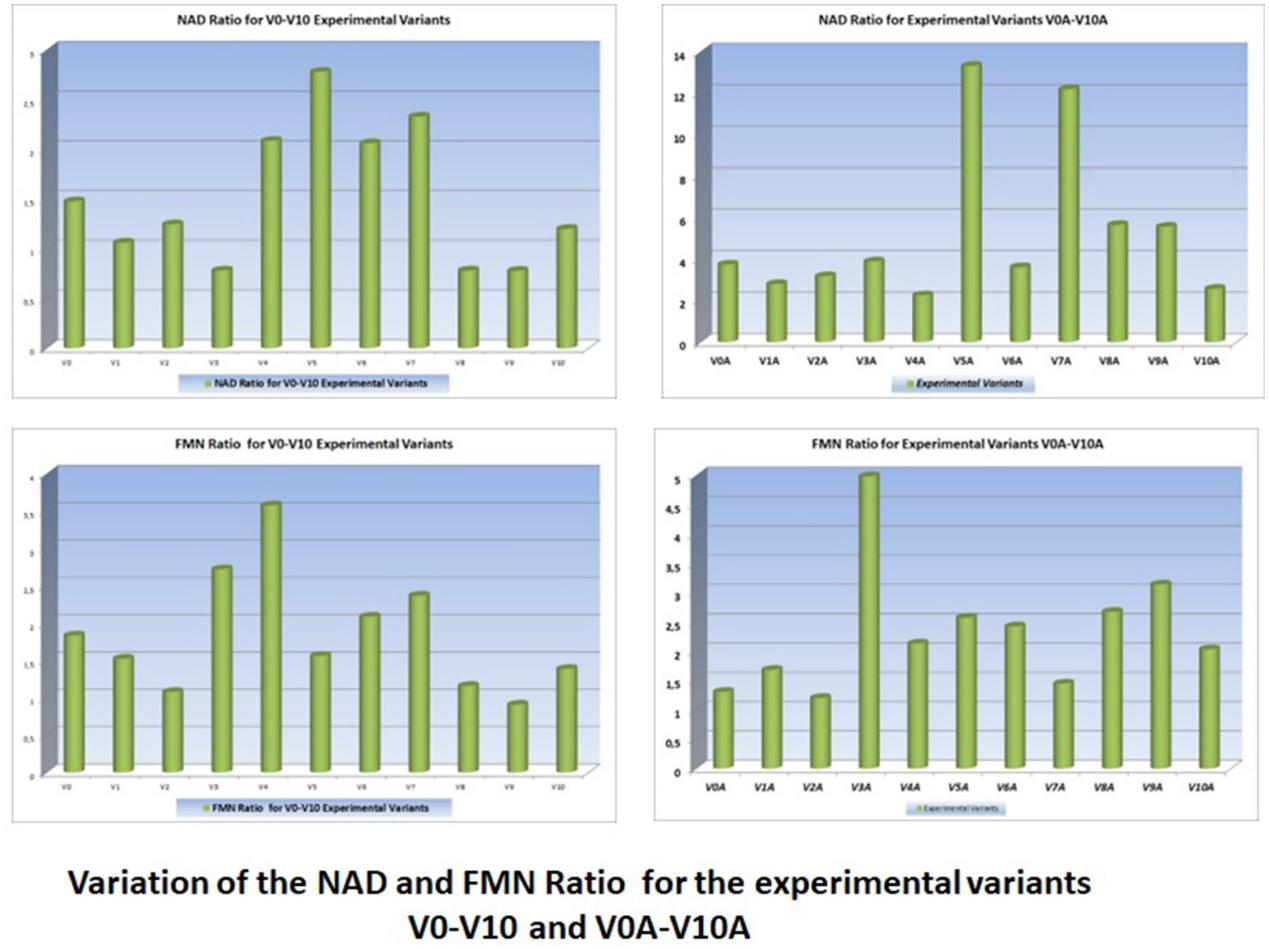
Variation of the NAD/NADH+H^+^ and FMN/FMNH+H^+^ ratios for V_0_-V_10_ and V_0A_-V_10A_ experimental variants (Supplementary Figures 1–6).

The concentration ratio of oxidized and reduced forms of these bio-compounds is extremely important for the health of consumers (Figure 3).

**Figure 4:**
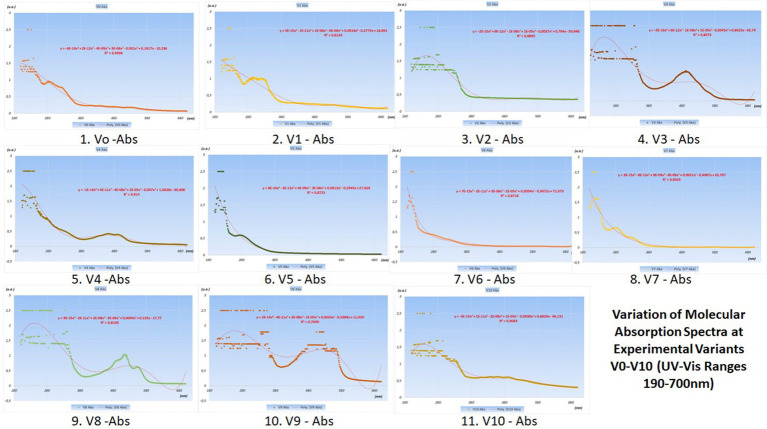
Variation of molecular absorption spectra (units absorbance) for V_0_-V_10_ experimental variants in UV (190–400 nm) and Vis (400–700 nm) Ranges.

**Figure 5:**
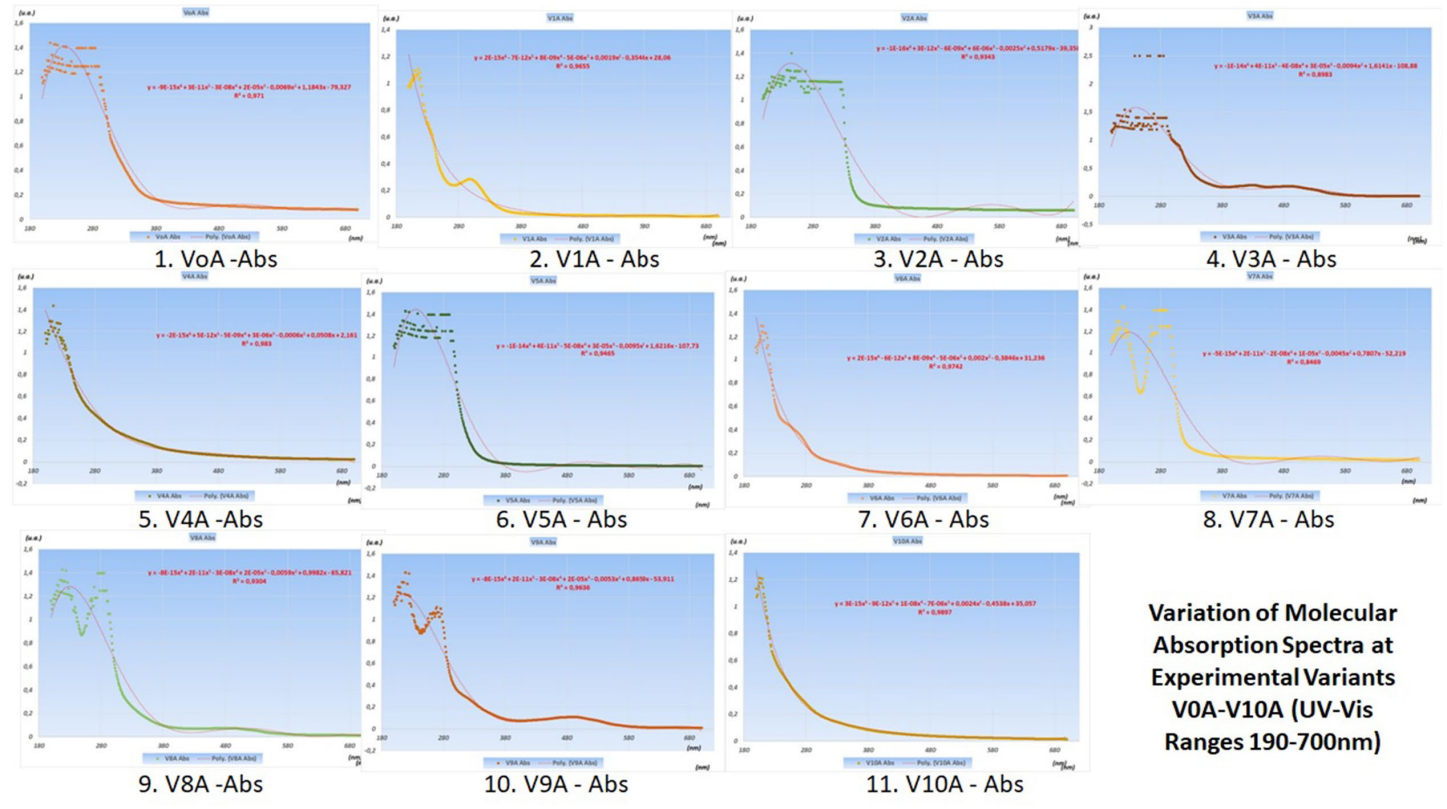
Variation of molecular absorption spectra (units absorbance) for V_0A_-V_10A_ experimental variants in UV (190–400 nm) and Vis (400–700 nm) ranges (Supplementary Figures 1–6).

The variations in their concentrations following the addition of various food additives are presented in the graphs with UV–Vis spectra in Figure 4 (juices from V0 variants) and Figure 5 (juices made from V0A variants).

The best juices with additives are considered to be those that induce the smallest changes in the curves of the molecular absorption spectra compared to the similar ones of the reference variants (no additives) from which they originate.

**Figure 6:**
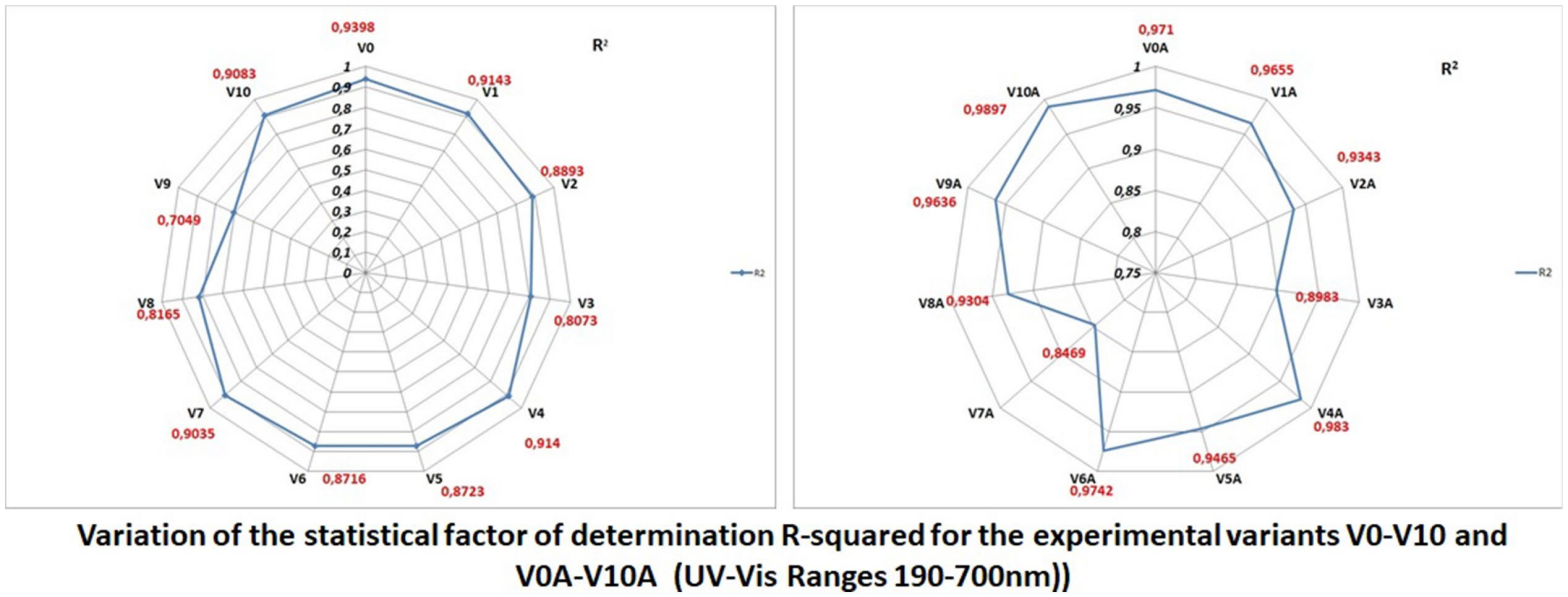
Variation of the squared-R at V_0_-V_10_ and V_0A_-V_10A_ experimental variants (in UV–Vis ranges: 190–700 nm).

To precisely highlight these minimal differences between the spectral curves of the additive and non-additive variants, pairwise statistical tests were used, with the help of Origin Pro 2020 software. In this article, only radar charts were used for the coefficients of determination squared R (Figure 6).

The best variants proposed for the next product series were obtained by comparing the spectral curves of Variants V_1_ through V_10_ with that of the witness variant V_0_, and V_1A_ through V_10A_ variants, respectively, with that of the V_0A_ variant.

The concentrations of bioactive compounds in the experimental V0 (pressed carrot juice) are higher than in V0A. This is normal, as V0A was obtained from pressed carrot residues. These carrot residues were diffused into drinking water and activated in the plasma field.

From the analysis of the graphs presented in Figures 1–6, several aspects can be highlighted:

- Preservatives, colorants, and sweeteners significantly alter the concentrations of the oxidized and reduced active forms of the main compounds in carrots (from Figures 1, 2);
- Preservatives affect the color of carrot juices, and therefore it is necessary to add colorants (beta-carotene or carmine). Carmine has proven protective activity, increasing resistance against the attack of sugar on some valuable compounds in carrot juice (synergistic effect in this case);
- From the analysis of V_0_, V_1_, and V_2_ variants, preservatives are shown to retain higher concentrations of active forms of FMNH2 and FMN, exhibiting a synergistic effect when used;
- Sugar consumes a significant portion of the concentrations of the active forms of FMNH2 and FMN, a fact resulting from the comparative analysis of V_0_ with V_5_. Even the natural stevioside (a natural sweetener from *Stevia rebaudiana*) influences the concentrations of the active forms of FMNH2 and FMN;
- From the analysis of the fructose-sweetened variant (a synthetic sweetener used), it is observed that fructose (V_7_) greatly reduced the concentrations of active forms of bio-compounds involved in redox processes;
- Very important and interesting is the coloring effect and preservation of the active forms, even under the action of sweeteners, of the carmine dye (a finding resulting from the comparative analysis of pairs of data from V_5_ with V_8_ and V_6_ with V_9_).
- The combinations of additives in V_9_ are superior in their effects to those in V_8_, with stevioside consuming fewer active forms of redox agents than beet sugar;
- Concentrations of active forms of NAD and NADH2 are sustained by the activity of the red-carmine dye, which greatly influences the redox balances in the juice;
- In the case of variants obtained using the activation method (from V_0A_ to V_10A_), carmine dye protects most concentrations of active principles involved in redox processes;
- Without the support of the polysaccharides in the juice (lignin, cellulose, and hemicellulose being removed during the processing method of activation), the concentrations of oxidized and reduced forms of NAD and NADH2 are significantly reduced under the action of acetic acid (V1A). After the countercurrent extraction of bioactive compounds from the pressed carrot residue, a primary carrot juice was obtained. This primary juice (which also contained soluble polysaccharides) was mixed with calcium hydroxide (lime) for 2 h. Then, carbon dioxide is bubbled through the mixture to neutralize the alkaline solution, forming calcium carbonate, and the mixture is filtered. This removes several polysaccharides that would have disturbed the final product design format of the carrot juice—functional food;
- Beet sugar reduces concentrations of active forms of bio-compounds (V_5A_);
- Even though it does not produce fermentation and is a hypocaloric sweetener, stevioside reduces the concentrations of active forms of vitamin A, whether reduced or oxidized (as seen in V_6A_).

The concentrations of reduced and oxidized forms of NAD and NADH2, respectively FMN and FMN H2 - as well as the ratio of these concentrations - are very important for the final redox potential (Figure 3).

- Product series 2 (carrot juice with additives) is superior to the series of products from which it derives (Series 1 from V_0_, juice without additives).
- To obtain superior quality and sensory products, it is necessary to add carrot juices obtained by pressing (variant V_0_) or through advanced processing (variant V_0A_).
- To obtain the best additive variants (those that least modify the chemical composition of the basic variants V_0_ and V_0A_), elements of mathematical statistics will be used.

From the statistical analysis, comparative—in pairs—of the variants of juices V_1_–V_10_, reported to V_0_ (Witness Variant), the best experimental variants were V_1_ (with few additives) and V_10_ (better sensorial but high additive—presented in File 2” Statistics, Supplementary materials 2, details”).

From the statistical analysis, comparative—in pairs—of the variants of juices V1A–V10A, reported to V0A (Witness Variant), the best experimental variants were V1A (with few additives) and V9A (better sensorial but high additive).

The Radar Plots (from Figure 6) show the differences between the R-squared statistical determination coefficients (determined as properties of the trend lines of the molecular absorption spectra curves specific to the experimental variants—shown in Figures 4, 5).

### 3.2 Determination of DNA changes induced by the attack of pathogenic microorganisms

Using the DNA changes recorded by the DNA/Proteid Biology Chipset DP200307, it was possible to determine, long before it was microscopically or visually detected, the specific white mold attack on carrot leaves and roots.

To calibrate the Proteid Biology Chipset: after subsequent microscopy, it was determined that the attack of the microorganism that led to the decrease in Total Phenolic Content was caused by the Alternaria Blight mold. This mold forms fluffy colonies with septate mycelium and large conidia with longitudinal and transverse septa (pore-spores). When it attacks, it consumes some of the polyphenols in the carrot leaves, and it is estimated that 10 min are enough for complete mitosis of the mold spores, with an interval of 20–40 min between mitosis and the appearance of septa. Only then does the mold attack become visible. Until then, the DNA changes recorded by the mathematical coprocessor of the UV–Vis spectrometer can be used at the two maxima of molecular absorption (at wavelengths of 230 and 260 nm) to record the decrease of antioxidant content (total polyphenols).

The raw materials (carrots) used have not been attacked by pathogenic/parasitic microorganisms, and, of course, no DNA alterations have been recorded in the mathematical coprocessor of the T92 + Spectrophotometer (picture with T92 plus coprocessor).

### 3.3 Atomic absorption spectroscopy

Using standardized and laboratory-developed methods (with their own Editing and Revision Procedures), the results are shown in Table 2 (which includes the main compositional and mineral characteristics of carrots, *Daucus carota L.*) and Table 3 (which includes data regarding the mineral characteristics of Romanian carrots, *Daucus carota* subsp. *carota*). These raw materials are particularly important, as the antioxidants in these plants are used (in the form of extracts) as food additives in the design and construction of the final food supplement.

**Table 2:** Atomic absorption spectroscopy for *Daucus carota* L. baseline.

Indicator/Constituent	Carrots (*Daucus carota* L.)	
	Dry Matter ppm (mg/kg) *	Aqueous extract 1:10 ppm (mg/L) average value
Na^+^	424 ± 4.03	44.68
K^+^	2,416 ± 3.18	172.40
Ca ^2+^	480 ± 1.64	5.49
Mg ^2+^	94 ± 0.65	2.29
Zn^2+^	2.4 ± 0.04	0.07
Mn ^2+^	216 ± 0.46	1.09
Fe ^2+^	22 ± 0.84	0.261
Al ^3+^	7.02 ± 0.06	0.557
Cu^2+^	0.2 ± 0.001	0.009
Pb^2+^	0.016 ± 0.001	Not detected

*Values are expressed as mean ± SD of triplicate measurements.

**Table 3:** Indicators for carrots used in Romanian food supplements.

Number	Indicators	Value*	Unit of measurement
1	Moisture	86 ± 1.2	(%)
2	Raw ash	1.2 ± 0.08	(% dry substance)
3	Crude protein	0.9 ± 0.02	(% dry substance)
4	Crude fat	0.32 ± 0.06	(% dry substance)
5	Total sugar	7.2 ± 0.04	(% dry substance)
6	Total fiber	1.84 ± 0.24	(% dry substance)
7	Ca ^2+^	481.42 ± 6.28	(mg/100 g)
8	Mg ^2+^	96.40 ± 3.64	(mg/100 g)
9	Na^+^	44.70 ± 6.42	(mg/100 g)
10	K^+^	2240.00 ± 46.24	(mg/100 g)
11	Cu^2+^	0.42 ± 0.06	(mg/100 g)
12	Zn^2+^	3.64 ± 0.48	(mg/100 g)

*Values are expressed as mean ± SD of triplicate measurements.

As can be seen from Tables 2, 3, no heavy metal pollution was recorded in the raw material samples used in the experiment.

Table 3 shows the main physical–chemical indicators of partially processed carrots, which enable the extraction of antioxidants used in the design of the food supplement. For valuable dietary supplements or functional foods, processed carrots should be high in potassium, calcium, and magnesium ions, have high turgidity (high water content), and be low in fiber and protein.

The minerals from the carrots (raw material) were analyzed using Atomic Absorption Spectrometry (see section 2.3). The mineral composition of the carrots is shown in Table 2.

### 3.4 Use of plasma field to improve supercritical fluid extraction technique

The extraction of carotenes with high antioxidant value was accelerated and enhanced by the combined field generated and maintained (throughout the extraction period) by the bio-membranes loaded in the field. For each product variant subjected to extraction (in this case, carrot pomace after pressing and juice removal), the determination of the optimal extraction pressure under supercritical and enhanced field conditions is performed by selection. Thus, the molecular absorption spectra recorded from the extract were compared with the molecular absorption spectra obtained at scales of known concentration of antioxidant provitamins in carrots (carotenes, lycopene). The pressure used in supercritical extraction was considered “optimal” when extracts with the highest carotenoid concentrations were obtained.

Using plant bio-membranes loaded in a modified carbon dioxide field produced a 20% increase in SFE yield when carbon dioxide at 31–32°C and 150–230 bar was used as a supercritical extraction agent (59, 68).

In order to achieve the objectives of the study (analysis of synergistic effects produced by certain antioxidants in specific functional foods), specialized supercritical extraction (SFE) techniques had to be employed, along with special techniques utilizing plasma-field activated membranes (to improve extraction yield, recovery, and utilization of valuable compounds from carrots) and methods for activating extracted compounds. This multifaceted approach has yielded several bioactive compounds with antioxidant properties, paving the way for their utilization in the production of competitive food supplements and functional foods (File 3 “Innovative Technology,” Supplementary materials 2, details”).

### 3.5 Use the plasmatic field to protect the food against pathogenic microbial attack in carrots

By using a simple microscopy technique, it was possible to photograph and film how the combined inductive field acts and how the antioxidant compounds in the natural juice (enriched with carotenoids in the activated extract) function. The photographs show the protective antioxidant effect of this juice at the cellular level. The inactivated carrot juice protected by this field also retains its bromatological properties unchanged for up to 48 h (without refrigeration under normal temperature and pressure conditions). It is noteworthy that when only pressed carrot juice was used, these protective and preservative activities against oxidation were not recorded. This demonstrates an increased antioxidant capacity of the bio-compounds in the juice selected for the analysis.

Furthermore, Supplementary Figure 4 presents images in which the combined field neutralizes and inactivates a microorganism introduced into the medium. It can be clearly distinguished how, as the field approaches, the microorganism activates its own defense system but is unable to resist the inductive effect of the field.

The use of equipment capable of producing a mixed field (cold plasma, magnetic, and gravitational) can protect food from contamination by microbial pathogens. An example of this is shown in Supplementary Figure 4, which illustrates a bactericidal effect of the field force line (the blue curved line in the images) on bacteria such as *Escherichia coli*. In less than 3 min, all the medium under test was free of pathogenic bacteria, as verified by repeatability and reproducibility.

## 4 Conclusion

- To obtain products with high antioxidant potential from carrots, it is necessary to use innovative technologies, especially for products derived from the waste resulting from the processing of carrots. The proposed innovative technologies led to an increased yield of extraction, recovery, and utilization of valuable antioxidants from carrots, as well as their stabilization in reaction kinetics (with food additives suggested for use to enhance the sensory properties of the final functional food). Synergistic effects are thus developed that improve the nutritional density of the final juices and their storage stability;
- Understanding the changes in the concentration ratios of active forms of NAD and NADH+H^+^ is very important for the study of the redox processes in carrot juices and their antioxidant capacity;
- Understanding the changes in the concentration ratios of active forms of FMN and FMNH+H^+^ is very important for the study of redox processes at the interface of carrot juices and air, and especially for improving the storage period (synergistic effect between V_1_ and V_10_);
- From this complex study of the variants of juices V_1_–V_10_, reported to V_0_ (Witness Variant), the best experimental variants were V_1_ (with few additives) and V_10_ (better sensory, but high additive).
- From this complex study of the variants of juices V_1A_–V_10A_, reported to V_0A_ (Witness Variant), the best experimental variants were V_1_ (with few additives) and V_9A_ (better sensory, but high additive).
- Using the techniques described in the article, a number of technological advantages can be achieved:
- Increased antioxidant capacity by promoting combined fields that produce preservation and conservation effects on the properties of antioxidant bio-compounds in food;
- The possibility of blocking the activity of potentially pathogenic, contaminating micro-organisms;
- Their use in the design and development of valuable food supplements from existing natural flora with raw materials of high antioxidant potential;
- Development of food additive management, taking into account the preservation of the antioxidant capacity of the final products and the synergism/antagonism of certain food additives;
- Promotion of manufacturing recipes that consider the preservation of the active antioxidant capacity of compounds and product bromatology;
- Realization of advanced product design and extended product shelf life (under normal conditions of temperature, humidity, and pressure);
- The use of devices such as combined field generators in holding/storage rooms can lead to significant cost savings by optimizing technological costs.

## Data Availability

The original contributions presented in the study are included in the article/Supplementary material, further inquiries can be directed to the corresponding authors.
