# Supplementary material for: Synergistic effects produced by certain antioxidants in valuable functional foods from the Romanian markets
Source: Front Nutr. 2025 Jun 19;12:1558597. doi: 10.3389/fnut.2025.1558597 (PMC12221894; doi:10.3389/fnut.2025.1558597)
Supplement: Supplementary file 1 [file Image_1.pdf]

Supplementary Material – Petre SĂVESCU – University of Craiova, Romania

$$E = E^0 + \frac{RT}{nF} \ln \frac{[\mathrm{Ox}]}{[\mathrm{Red}]}$$

Figure 1 – Nernst Equation *–the logarithm of the ratio of the concentrations of the oxidized and reduced forms defines the redox potential of the reaction medium – especially when NAD- or FMN-dependent oxidoreductases are active. Therefore, the ratios (NAD)/(NADH+H$^+$) and (FMN)/(FMNH+H$^+$) are very important*

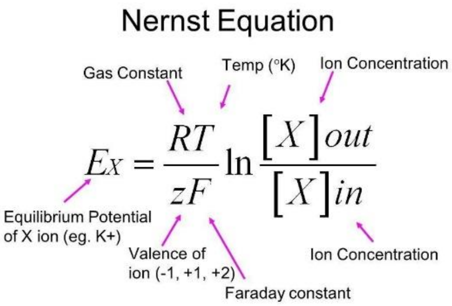

Figure 1 a- explaining the indicators in the Nernst Equation

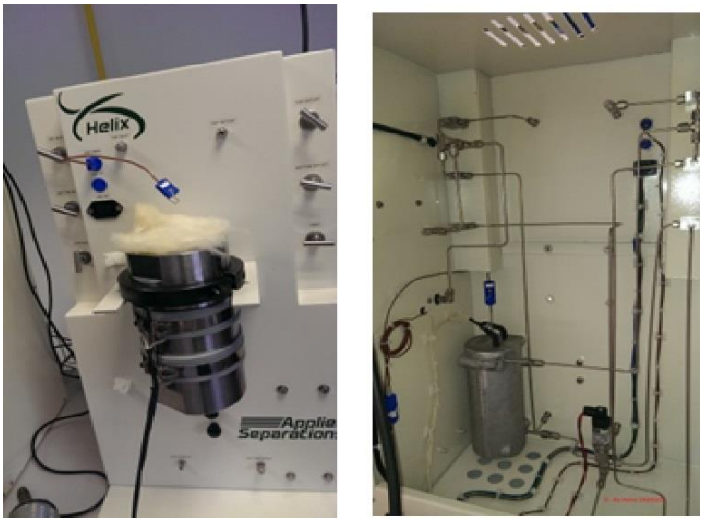

Figure 2- Helix Natural Products SFE Unit with activated bio-membranes

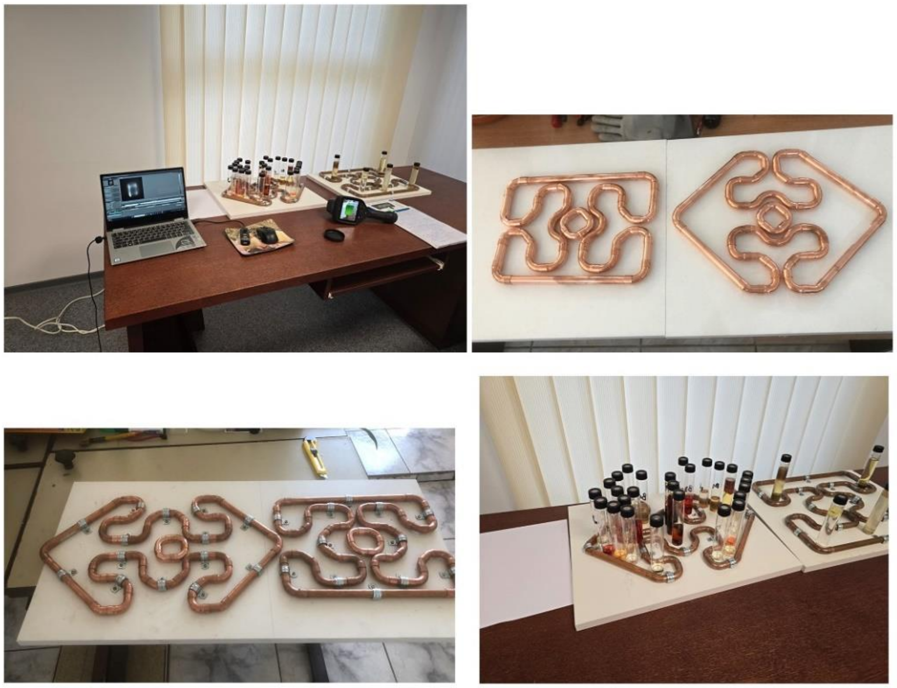

Figure 3 – The used field generators for active the experimental Variants V_0A-$V_{10}A$

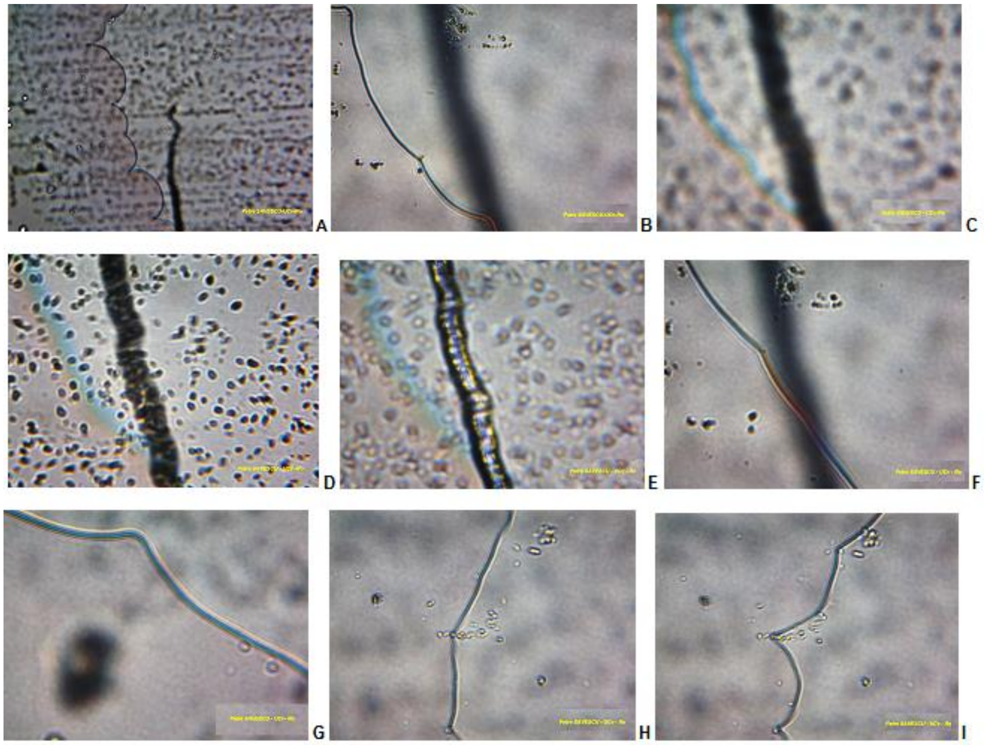

Figure 4 - Antimicrobial activity of the mixed field - case study destruction of Escherichia Coli bacteria

Image A: The mixed field force line (the blue line at the edge of the pink field) approaches the Escherichia Coli Bacteria (the black bacillus); When approaching the bacteria, the mixed field force increases in intensity (the intense blue color as it appears in image C starting from the status in image B); in figure D – the bacteria goes on the defensive upon contact with the mixed field line; in image E- the bacteria's defense system is in a state of maximum activation (the entire bacterium exhibits a certain fluorescence); F- The last contact between Bacteria and Field: the force line briefly acquires a rusty color upon contact, E Coli has lost the fight and turns into a lifeless, black amorphous mass; in images H and I the mixed field continues its course in the environment decontaminated by bacteria

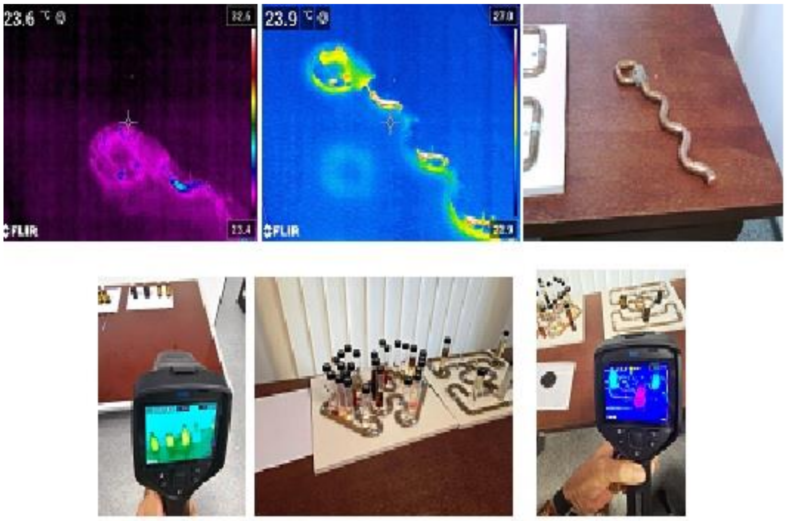

Figure 5 – Charging of field and activated experimental variants

The plasma field charging of the solutions can be done both with specific generators and with the help of amplifier rods. The charging can also be highlighted with the help of FLIR E86 thermal imaging cameras.

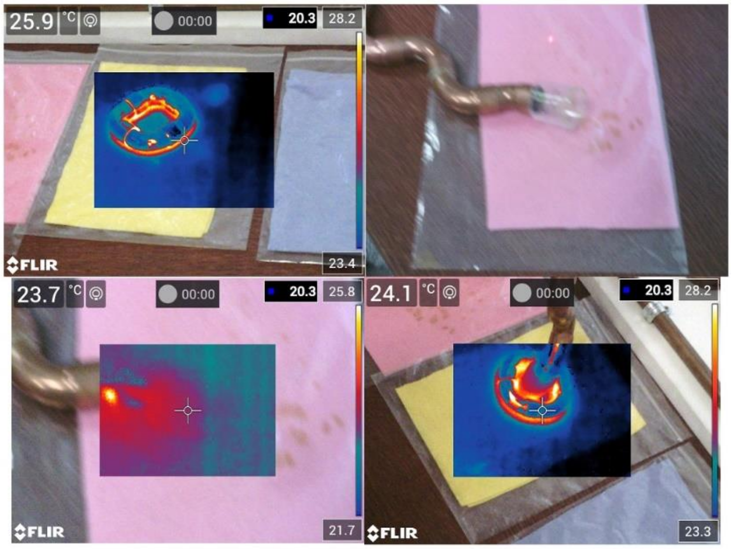

Figure 6 - Charging of bio-membranes

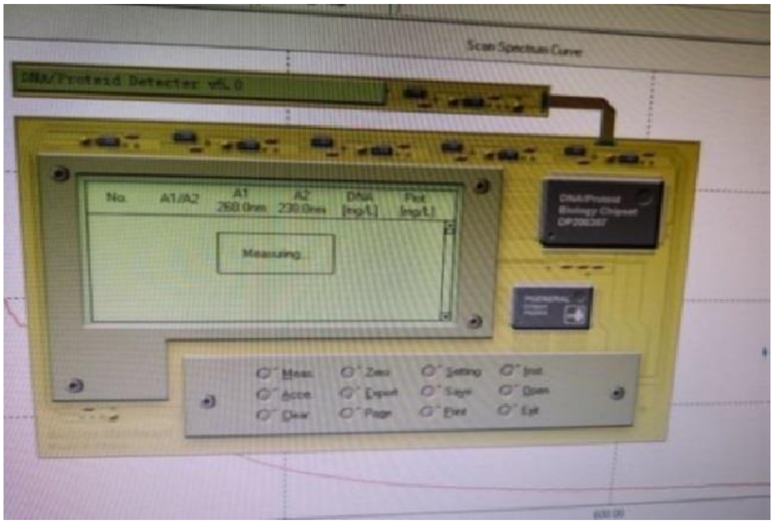

Figure 7 – DNA Proteid Chipset
