# Supplementary material for: Synergistic effects produced by certain antioxidants in valuable functional foods from the Romanian markets
Source: Front Nutr. 2025 Jun 19;12:1558597. doi: 10.3389/fnut.2025.1558597 (PMC12221894; doi:10.3389/fnut.2025.1558597)
Supplement: Supplementary file 2 [file Data_Sheet_1.pdf]

Subsection -Preparation of samples. Sources of carrots

The carrots used in the production of the juice were grown in a vegetable farm in South-Western Romania (Oltenia), in an area recognized for the quality of the soil (cernoziom) rich in minerals and growth factors. The vegetable farm is currently being converted to organic farming.

Romanian carrot varieties were chosen because of their particular genetics and high concentrations of type A provitamins (β-carotene and lycopene). The drinking water used was also controlled so as not to produce a change in ionic or mineral concentration. It is known that over 1/3 of the therapeutic and mineral waters in the EU are in Romania. Also, due to past underfunding, most of the agricultural land in Romania was less treated with synthetic fertilizers or pesticides.

Trace analysis for heavy metals and minerals (by Atomic Absorption Spectrometry-A.A.S.) was necessary to be certain that these undesirable substances were absent in the final carrot juice.

Carrots were thoroughly washed (with potable water) and peeled (following a specific laboratory procedure).

A. Before the physical, chemical, microbiological tests, the samples were checked (samples were representative, not altered during storage and/or transportation. Before taking a sample for analysis, each sample was homogenized.

B. For the analyses involving UV-VIS molecular absorption spectrometric measurements, the absorbance of the sample was measured relative to that of the control sample (with double-distilled water). These measurements were carried out - at the wavelengths at which the absorbance maxima for each compound were recorded in the T92+ spectrophotometer (at 10 mm optical path) - on the basis of the calibration curve.

C. The results were calculated using calibration curves prepared with standard solutions at various dilutions. A coordinate system was plotted - with the absorbance values of the standard solutions on the ordinate (Oy) and the absorbance values of the compounds to be determined on the abscissa (Ox). A linear equation was thus obtained. Using the "Single Addition Method" and Certified Reference Materials from Merck it was possible to find the wavelength at which the maximum molecular absorbance of the valuable biocompounds in carrot juices (normal variants V0-V10 and activated variants - functional food V0A-V10A) was maximized.

D. The molecular absorbances (a.u.) were then read on the T92+ UV-VIS spectrophotometer. According to the Lambert-Beer Law, these absorbances are direct functions of concentration.

Eliminate or minimize risks:

In the case of *erroneous results* - risks were eliminated by reducing the volume of the sample analyzed and increasing the working (reading) range - where the samples were not completely clear.

To minimize/eliminate *measurement errors* - the risk was avoided by observing sample incubation times and conditions.

To prevent *the risk of contamination* - biodegradable detergent and hot water were used to clean the equipment/apparatus that were in contact with the samples, then rinsed with deionized water.

As regards the measurements of indicators (metals and minerals) carried out by Atomic Absorption Spectrometry (results obtained - presented in Table 2):

- As the principle of the method used, after mineralization of the sample in the microwave oven and dilution in the volumetric flask, the solution was aspirated into the flame of an atomic absorption spectrometer, using cavity cathode lamps for each metal analyzed. The metal concentration was proportional to the resulting absorbance.
- The concentrations of the indicators in carrots (Tables 2 and 3) could be obtained by F-AAS (Flame Atomic Absorption Spectroscopy), directly or after calcination at 550°C/microwave digestion.
- The method (for the determination of Ca,Mg,Na, K) applied on the matrix "carrot juice" had as reference documents:
- SR EN 15505: 2008 - Foodstuffs. Determination of trace elements. Determination of Na and Mg by FAAS after microwave digestion;
- SR EN 1134: 1996 - Fruit and vegetable juices. Determination of Na, K, Ca, Mg by AAS;
- SR EN 14084: 2003 - Foodstuffs. Determination of trace elements. Determination of Pb, Zn, Cu, Fe by AAS after microwave digestion;
- SR EN 13804: 2013 - Foodstuffs. Determination of elements and their chemical species.
- Lead required the use of GF-AAS (Graphite Furnace Atomic Absorption Spectroscopy).
- The choice of the optimal method to use (F-AAS or GF-AAS technique) was determined by the concentrations that were analyzed.

The solid samples used for the determinations - were ground in a homogenizer until a homogeneous paste was obtained.

Liquid samples of carrot juice were homogenized by repeated shaking of the container.

Only the edible parts of the carrots (without leaves or leaf sheaths) were investigated in the analyses.

When carrots were used, the ends, dirty and rotten parts were removed, scraped clean and washed. During washing of the samples, the extraction/solvation effect of compounds from the cut surfaces was avoided. The final rinsing was carried out using deionized water, and traces of the rinsing water were blotted with paper.

After calcination/mineralization of the samples, the direct analysis was performed (which was done in both ways: calibration with the standard solutions and checking two points of the curve, which had to be within the established acceptance range).

The calibration curves were stable and complied with the technical conditions of the equipment; thus they had R2 above 0.9950 and the characteristic concentrations of the respective curves (those appearing in the equipment cook-books) were ± 30% of the characteristic concentrations given by the equipment for the respective element. Failure to fall within this range would have indicated a technical problem with the equipment (voltage on the furnace, contact piece, burner problems).

The metal content of the samples was deduced from the calibration curves performed with at least 5 standards. For each element the linear range of the calibration (calibration) curve was determined, taking into account the dilution factor.

Wavelengths, gas mixtures, temperature programming, flame type air-acetylene, oxidizing, other F-AAS instrumental parameters were taken from the Equipment Technical Manual.

GF-AAS has been used for the determination of Lead. Here, microwave digestion can lead to relatively high dilutions of carrot juice samples; so the GF-AAS technique could also be used for the determination of Copper. In the case of Lead, matrix modifier (the one established in the

AAS Spectrometer User Manual, and an injection volume of 20 µL, with 4 temperature steps (120° /650° /1500° / 2300° C) with a ballistic temperature increase at short time intervals (10/10/4/4)seconds was used.

When calculating the results, the equipment automatically displayed the calibration (calibration) curve and the measurements for the direct sample in concentration units (mg/L for F-AAS).

The C-content was calculated as a function of the mass of the element to be determined, in mg/kg:

$$C= \frac{csol\ x\ V\ xF}{m}$$

Where, c_{sol} - the concentration of the sample solution (divided by 1000, for expression in mg/Kg

V- volume of the sample solution in mL
F-Sample dilution factor
m- sample mass in grams

The majority of the physical, chemical and microbiological tests were carried out in Romanian Testing Laboratories, laboratories accredited to EN ISO/IEC 17025:2018. ISO/IEC 17025 "General requirements for the competence of testing and calibration laboratories", is the international reference for testing and calibration laboratories wanting to demonstrate their capacity to deliver reliable results. Eliminating risks was made much easier by complying with the requirements of EN ISO/IEC 17025:2018.

In order to comply with Traceability Policy P-05 RENAR (Romanian Accreditation Association), Single Element CRM solution by Merck, in various concentrations for AAS (C.R.M. - Certified and Reference Material), was used for AAS. Any measurement is only as good as the reference standard used to calibrate the system. When the accuracy of your measurement matters, it is important to start every analysis with a fresh vial of the highest quality, certified reference or calibration standards.

These certified reference material standard solutions are produced from the highest purity starting material, fulfilling both ISO/IEC 17025 and ISO 17034 guidelines. The reference materials are ready-to-use solutions, certified and traceable to primary standard reference material from NIST. Plus, the specific and comprehensive documentation includes proper uncertainty calculations on the certificate of analysis issued according to ISO Guide 31 guidelines.
