# Supplementary material for: Synergistic effects produced by certain antioxidants in valuable functional foods from the Romanian markets
Source: Front Nutr. 2025 Jun 19;12:1558597. doi: 10.3389/fnut.2025.1558597 (PMC12221894; doi:10.3389/fnut.2025.1558597)
Supplement: Supplementary file 3 [file Data_Sheet_2.pdf]

Statistics 2 - Correlation coefficients and Pearson, Spearman and Kendal correlations

For the purpose of analyzing the obtained data and additional statistical interpretation, Origin Pro 2022 software was used.

To facilitate the analysis of the spreadsheets, the following correspondence matrix was used:

For Experimental Variants V_0-V_{10} ***Table 1***

Column	Equivalent	Column	Equivalent
A(X1)	Wavelength [nm]	G (YI)	Absorption V_5 [u.a.]
B(YI)	Absorption V_0 [u.a.]	H (Y1)	Absorption V_6 [u.a.]
C(Y1)	Absorption V_1 [u.a.]	I (Y1)	Absorption V_7 [u.a.]
D (Y1)	Absorption V_2 [u.a.]	J (Y1)	Absorption V_8 [u.a.]
E (Y1)	Absorption V_3 [u.a.]	K(Y1)	Absorption V_9 [u.a.]
F(Y1)	Absorption V_4 [u.a.]	L(Y1)	Absorption V_{10} [u.a.]

For Experimental Variants V_{0A}-V_{10A} ***Table 2***

Column	Equivalent	Column	Equivalent
A(X1)	Wavelength [nm]	G (YI)	Absorption V_{5A} [u.a.]
B(YI)	Absorption V_{0A} [u.a.]	H (Y1)	Absorption V_{6A}[u.a.]
C(Y1)	Absorption V_{1A} [u.a.]	I (Y1)	Absorption V_{7A}[u.a.]
D (Y1)	Absorption V_{2A} [u.a.]	J (Y1)	Absorption V_{8A}[u.a.]
E (Y1)	Absorption V_{3A} [u.a.]	K(Y1)	Absorption V_{9A}[u.a.]
F(Y1)	Absorption V_{4A} [u.a.]	L(Y1)	Absorption V_{10A} [u.a.]

Statistical calculation elements for normal variants V_0-V_{10} (based on molecular absorbance data in the UV range- 190 nm - 400 nm)

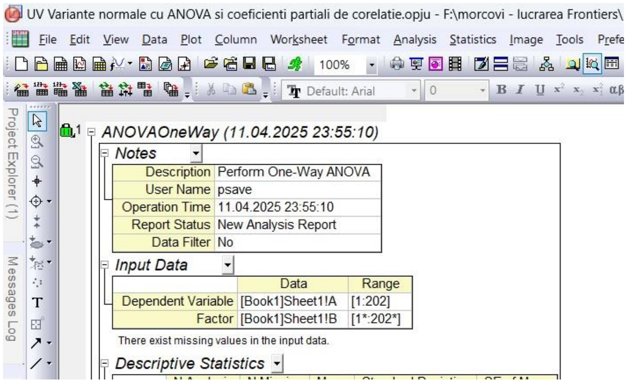

Figure 1 – Input Data for One Way ANOVA– Normal Experimental Variants in UV Range [190-400nm]

One Way ANOVA

Overall ANOVA

	DF	Sum of Squares	Mean Square	F Value	Prob>F
Model	196	676636,33333	3452,22619	216,89379	4,26797E-5
Error	4	63,66667	15,91667		
Total	200	676700			

Null Hypothesis: The means of all levels are equal.
Alternative Hypothesis: The means of one or more levels are different.
At the 0.05 level, the population means are significantly different.

Fit Statistics

R-Square	Coeff Var	Root MSE	Data Mean
0,99991	0,0133	3,98957	300

Means Comparisons

- Bonferroni Test
- Scheffe Test
- Tukey Test
- Sidak Test
- Fisher Test
- Bonholm Test
- Sidakholm Test

Sig equals 1 indicates that the difference of the means is significant at the 0,05 level.
Sig equals 0 indicates that the difference of the means is not significant at the 0,05 level.

Homogeneity of Variance Test

Sheet1 | ANOVA1Way1 | CorrCoef1 | Pearson1 | Spearman1 | Kendall1

Figure 2 – Overall One Way ANOVA– Normal Experimental Variants in UV Range [190-400nm]

1 Correlations Coefficients (12.04.2025 12:32:52)

Notes

X-Function	Correlations Coefficients
User Name	psave
Time	12.04.2025 12:32:52
Data Filter	No

Input Data

	Data	Range
A	[Book1]Sheet1!A	[1:202]
B	[Book1]Sheet1!B	[1:202]
C	[Book1]Sheet1!C	[1:202]
D	[Book1]Sheet1!D	[1:202]
E	[Book1]Sheet1!E	[1:202]
F	[Book1]Sheet1!F	[1:202]
G	[Book1]Sheet1!G	[1:202]
H	[Book1]Sheet1!H	[1:202]
I	[Book1]Sheet1!I	[1:202]
J	[Book1]Sheet1!J	[1:202]
K	[Book1]Sheet1!K	[1:202]
L	[Book1]Sheet1!L	[1:202]

Descriptive Statistics

	N	Mean	SD	Sum	Min	Max
A	201	300	58,16786	60300	200	400
B	201	3,34993	1,77012	673,3357	0,9083	9,999
C	201	3,6376	1,66782	731,1579	1,1175	9,999
D	201	4,61977	1,98976	928,5747	1,6706	9,999
E	201	5,90008	2,83027	1185,9165	1,6044	9,999
F	201	3,50011	2,58991	703,5227	0,9183	9,999
G	201	2,42827	2,41612	488,0816	0,3026	9,999
H	201	1,91896	2,08338	385,7102	0,2419	9,999
I	201	2,38863	2,23235	480,1155	0,1552	9,999
J	201	5,77969	2,84101	1161,7172	1,2	9,999
K	201	5,97162	2,16677	1200,2955	2,4787	9,999
L	201	4,60302	1,59242	925,2073	2,3205	9,999

Pearson Correlations

Figure 3 – Correlations Coefficients– Normal Experimental Variants in UV Range [190-400nm]

A(X)	B(Y)	C(Y)	D(Y)	E(Y)	F(Y)	G(Y)	H(Y)	I(Y)	J(Y)	K(Y)	L(Y)	M(Y)
	A	B	C	D	E	F	G	H	I	J	K	L
Pearson orrelations	Pearson Correlations	Pearson Correlations	Pearson Correlations	Pearson Correlations	Pearson Correlations	Pearson Correlations	Pearson Correlations	Pearson Correlations	Pearson Correlations	Pearson Correlations	Pearson Correlations	Pearson Correlations
	1	-0,92646	-0,88108	-0,78052	-0,69665	-0,88449	-0,82679	-0,77406	-0,86089	-0,74679	-0,44664	-0,83215
	-0,92646	1	0,91821	0,72973	0,72076	0,91539	0,85665	0,75601	0,85515	0,76177	0,50563	0,80639
	-0,88108	0,91821	1	0,74175	0,73074	0,86575	0,84765	0,7325	0,83048	0,76457	0,53291	0,82576
	-0,78052	0,72973	0,74175	1	0,84385	0,64101	0,53654	0,45718	0,56602	0,85857	0,60737	0,86818
	-0,69665	0,72076	0,73074	0,84385	1	0,60429	0,56118	0,47966	0,58542	0,94245	0,70193	0,82027
	-0,88449	0,91539	0,86575	0,64101	0,60429	1	0,92064	0,8605	0,92632	0,66322	0,42217	0,76372
	-0,82679	0,85665	0,84765	0,53654	0,56118	0,92064	1	0,92137	0,96422	0,61449	0,377	0,66527
	-0,77406	0,75601	0,7325	0,45718	0,47966	0,8605	0,92137	1	0,96285	0,54794	0,29241	0,57185
	-0,86089	0,85515	0,83048	0,56602	0,58542	0,92632	0,96422	0,96285	1	0,64596	0,37953	0,68947
	-0,74679	0,76177	0,76457	0,85857	0,94245	0,66322	0,61449	0,54794	0,64596	1	0,72636	0,84428
	-0,44664	0,50563	0,53291	0,60737	0,70193	0,42217	0,377	0,29241	0,37953	0,72636	1	0,61133
	-0,83215	0,80639	0,82576	0,86818	0,82027	0,76372	0,66527	0,57185	0,68947	0,84428	0,61133	1

Figure 4 – Pearson Correlations – Normal Experimental Variants in UV Range [190-400nm]

A(X)	B(Y)	C(Y)	D(Y)	E(Y)	F(Y)	G(Y)	H(Y)	I(Y)	J(Y)	K(Y)	L(Y)	M(Y)
	A	B	C	D	E	F	G	H	I	J	K	L
Spearman Correlations	Spearman Correlations	Spearman Correlations	Spearman Correlations	Spearman Correlations	Spearman Correlations	Spearman Correlations	Spearman Correlations	Spearman Correlations	Spearman Correlations	Spearman Correlations	Spearman Correlations	Spearman Correlations
A	1	-0,98453	-0,9043	-0,89699	-0,77935	-0,98853	-0,99016	-0,99828	-0,97888	-0,80196	-0,52746	-0,9186
B	-0,98453	1	0,93467	0,91277	0,82531	0,98696	0,98466	0,98636	0,97645	0,84379	0,57417	0,93994
C	-0,9043	0,93467	1	0,8015	0,77656	0,9066	0,90842	0,9067	0,89555	0,77841	0,53038	0,8426
D	-0,89699	0,91277	0,8015	1	0,88894	0,91498	0,90661	0,90396	0,90129	0,89255	0,6229	0,95802
E	-0,77935	0,82531	0,77656	0,88894	1	0,80254	0,80165	0,78778	0,80887	0,94113	0,67163	0,87623
F	-0,98853	0,98696	0,9066	0,91498	0,80254	1	0,98818	0,99186	0,97657	0,81965	0,55891	0,93399
G	-0,99016	0,98466	0,90842	0,90661	0,80165	0,98818	1	0,9927	0,99229	0,82015	0,54943	0,93361
H	-0,99828	0,98636	0,9067	0,90396	0,78778	0,99186	0,9927	1	0,98168	0,80975	0,5372	0,924
I	-0,97888	0,97645	0,89555	0,90129	0,80887	0,97657	0,99229	0,98168	1	0,82744	0,55927	0,92702
J	-0,80196	0,84379	0,77841	0,89255	0,94113	0,81965	0,82015	0,80975	0,82744	1	0,68692	0,88965
K	-0,52746	0,57417	0,53038	0,6229	0,67163	0,55891	0,54943	0,5372	0,55927	0,68692	1	0,6116
L	-0,9186	0,93994	0,8426	0,95802	0,87623	0,93399	0,93361	0,924	0,92702	0,88965	0,6116	1

Figure 5 – Spearman Correlations – Normal Experimental Variants in UV Range [190-400nm]

A(X)	B(Y)	C(Y)	D(Y)	E(Y)	F(Y)	G(Y)	H(Y)	I(Y)	J(Y)	K(Y)	L(Y)	M(Y)
	A	B	C	D	E	F	G	H	I	J	K	L
Kendall Correlations	Kendall Correlations	Kendall Correlations	Kendall Correlations	Kendall Correlations	Kendall Correlations	Kendall Correlations	Kendall Correlations	Kendall Correlations	Kendall Correlations	Kendall Correlations	Kendall Correlations	Kendall Correlations
A	1	-0,92472	-0,78329	-0,79797	-0,66204	-0,9401	-0,94339	-0,98599	-0,90895	-0,68396	-0,45042	-0,81357
B	-0,92472	1	0,84154	0,81737	0,71432	0,92621	0,93024	0,93377	0,90689	0,73162	0,4881	0,84138
C	-0,78329	0,84154	1	0,68948	0,64982	0,784	0,8017	0,79428	0,78383	0,65983	0,42663	0,71981
D	-0,79797	0,81737	0,68948	1	0,79043	0,80763	0,805	0,80858	0,79323	0,78955	0,5226	0,87632
E	-0,66204	0,71432	0,64982	0,79043	1	0,68711	0,69424	0,67287	0,69917	0,89189	0,56131	0,76555
F	-0,9401	0,92621	0,784	0,80763	0,68711	1	0,93051	0,95044	0,89491	0,70425	0,4675	0,83173
G	-0,94339	0,93024	0,8017	0,805	0,69424	0,93051	1	0,95655	0,95516	0,71241	0,46416	0,83143
H	-0,98599	0,93377	0,79428	0,80858	0,67287	0,95044	0,95655	1	0,92253	0,69463	0,46049	0,82458
I	-0,90895	0,90689	0,78383	0,79323	0,69917	0,89491	0,95516	0,92253	1	0,71872	0,46513	0,8201
J	-0,68396	0,73162	0,65983	0,78955	0,89189	0,70425	0,71241	0,69463	0,71872	1	0,57131	0,7779
K	-0,45042	0,4881	0,42663	0,5226	0,56131	0,4675	0,46416	0,46049	0,46513	0,57131	1	0,50641
L	-0,81357	0,84138	0,71981	0,87632	0,76555	0,83173	0,83143	0,82458	0,8201	0,7779	0,50641	1

Figure 6 – Kendall Correlations – Normal Experimental Variants in UV Range [190-400nm]

Statistical calculation elements for normal variants V_0-V_{10} (based on molecular absorbance data in the Visible range, 400 nm-700 nm)

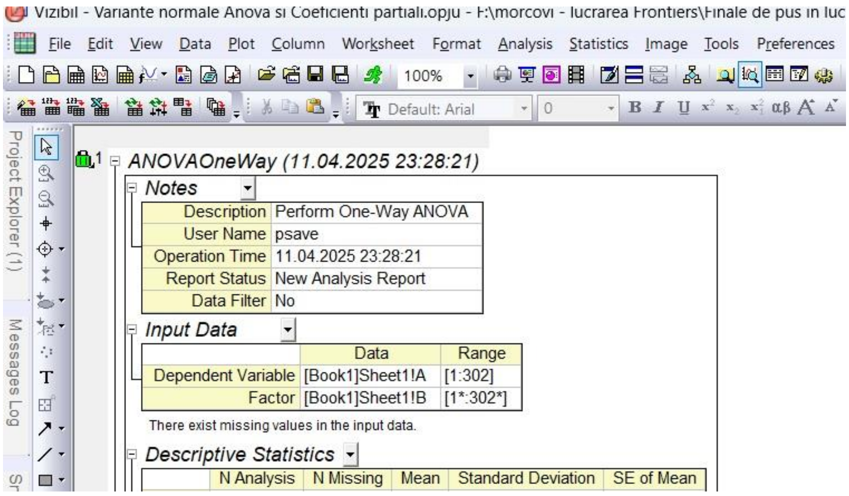

Figure 7 – Input Data for ANOVA One Way – Normal Experimental Variants in VIS Range [400-700nm]

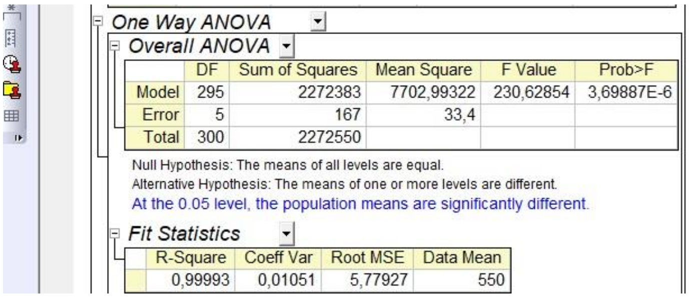

Figure 8 – Overall One Way ANOVA– Normal Experimental Variants in VIS Range [400-700nm]

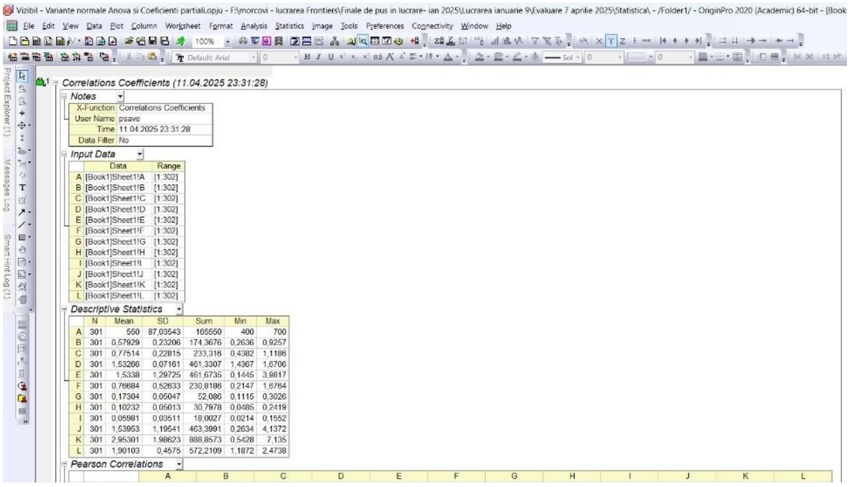

Vizibil - Variante normale Anova si Coeficienti partiali.opju - F:\morcovi - lucrarea Frontiers\Finale de pus in lucrare- ian 2025\Lucrarea ianuarie 9\Evaluare 7 aprilie 2025\Statistica\ - /Folder1/ - OriginPro 2020 (Academic) 64-bit - [Book

File Edit View Data Plot Column Worksheet Format Analysis Statistics Image Tools Preferences Connectivity Window Help

Correlations Coefficients (11.04.2025 23:31:28)

Notes

X-Function	Correlations Coefficients
User Name	psave
Time	11.04.2025 23:31:28
Data Filter	No

Input Data

	Data	Range
A	[Book1]Sheet1!A	[1:302]
B	[Book1]Sheet1!B	[1:302]
C	[Book1]Sheet1!C	[1:302]
D	[Book1]Sheet1!D	[1:302]
E	[Book1]Sheet1!E	[1:302]
F	[Book1]Sheet1!F	[1:302]
G	[Book1]Sheet1!G	[1:302]
H	[Book1]Sheet1!H	[1:302]
I	[Book1]Sheet1!I	[1:302]
J	[Book1]Sheet1!J	[1:302]
K	[Book1]Sheet1!K	[1:302]
L	[Book1]Sheet1!L	[1:302]

Descriptive Statistics

	N	Mean	SD	Sum	Min	Max
A	301	550	87,03543	165550	400	700
B	301	0,57929	0,23206	174,3676	0,2636	0,9257
C	301	0,77514	0,22815	233,316	0,4382	1,1186
D	301	1,53266	0,07161	461,3307	1,4367	1,6706
E	301	1,5338	1,29725	461,6735	0,1445	3,9817
F	301	0,76684	0,52633	230,8186	0,2147	1,6764
G	301	0,17304	0,05047	52,086	0,1115	0,3026
H	301	0,10232	0,05013	30,7978	0,0485	0,2419
I	301	0,05981	0,03511	18,0027	0,0214	0,1552
J	301	1,53953	1,19541	463,3991	0,2634	4,1372
K	301	2,95301	1,98623	888,8573	0,5428	7,135
L	301	1,90103	0,4575	572,2109	1,1872	2,4738

Pearson Correlations

		A	B	C	D	E	F	G	H	I	J	K	L

Figure 9 – Correlations Coefficients– Normal Experimental Variants in VIS Range [400-700nm]

A(X)	B(Y)	C(Y)	D(Y)	E(Y)	F(Y)	G(Y)	H(Y)	I(Y)	J(Y)	K(Y)	L(Y)	M(Y)
	A	B	C	D	E	F	G	H	I	J	K	L
Pearson Correlations	Pearson Correlations	Pearson Correlations	Pearson Correlations	Pearson Correlations	Pearson Correlations	Pearson Correlations	Pearson Correlations	Pearson Correlations	Pearson Correlations	Pearson Correlations	Pearson Correlations	Pearson Correlations
A	1	-0,98367	-0,99123	-0,99127	-0,77092	-0,86598	-0,96126	-0,93504	-0,94518	-0,60949	-0,66259	-0,97215
B	-0,98367	1	0,9986	0,9881	0,82479	0,88261	0,94118	0,91319	0,92885	0,67882	0,72392	0,98231
C	-0,99123	0,9986	1	0,99057	0,81596	0,87902	0,94579	0,91719	0,93189	0,67015	0,71844	0,98405
D	-0,99127	0,9881	0,99057	1	0,75608	0,86336	0,98082	0,96234	0,97178	0,58212	0,62988	0,95994
E	-0,77092	0,82479	0,81596	0,75608	1	0,9007	0,63727	0,58131	0,60831	0,901	0,88482	0,89124
F	-0,86598	0,88261	0,87902	0,86336	0,9007	1	0,80559	0,76707	0,77898	0,65594	0,67241	0,93141
G	-0,96126	0,94118	0,94579	0,98082	0,63727	0,80559	1	0,99642	0,998	0,4293	0,47869	0,89299
H	-0,93504	0,91319	0,91719	0,96234	0,58131	0,76707	0,99642	1	0,99899	0,36696	0,41488	0,85323
I	-0,94518	0,92885	0,93189	0,97178	0,60831	0,77898	0,998	0,99899	1	0,40258	0,45046	0,87058
J	-0,60949	0,67882	0,67015	0,58212	0,901	0,65594	0,4293	0,36696	0,40258	1	0,96877	0,73923
K	-0,66259	0,72392	0,71844	0,62988	0,88482	0,67241	0,47869	0,41488	0,45046	0,96877	1	0,77902
L	-0,97215	0,98231	0,98405	0,95994	0,89124	0,93141	0,89299	0,85323	0,87058	0,73923	0,77902	1

Figure 10 – Pearson Correlations – Normal Experimental Variants in VIS Range [400-700nm]

A(X)	B(Y)	C(Y)	D(Y)	E(Y)	F(Y)	G(Y)	H(Y)	I(Y)	J(Y)	K(Y)	L(Y)	M(Y)
	A	B	C	D	E	F	G	H	I	J	K	L
Spearman Correlations	Spearman Correlations	Spearman Correlations	Spearman Correlations	Spearman Correlations	Spearman Correlations	Spearman Correlations	Spearman Correlations	Spearman Correlations	Spearman Correlations	Spearman Correlations	Spearman Correlations	Spearman Correlations
A	1	-0,9963	-0,99913	-0,99985	-0,83223	-0,92109	-1	-1	-0,99994	-0,69629	-0,6798	-0,95369
B	-0,9963	1	0,99807	0,9963	0,82938	0,91793	0,9963	0,9963	0,99625	0,69525	0,67982	0,95565
C	-0,99913	0,99807	1	0,99907	0,83161	0,92058	0,99912	0,99912	0,99907	0,696	0,67974	0,95474
D	-0,99985	0,9963	0,99907	1	0,83218	0,92103	0,99985	0,99985	0,99977	0,69622	0,68003	0,95382
E	-0,83223	0,82938	0,83161	0,83218	1	0,95016	0,83223	0,83223	0,83216	0,932	0,91605	0,90328
F	-0,92109	0,91793	0,92058	0,92103	0,95016	1	0,92109	0,92109	0,92103	0,81469	0,7996	0,98014
G	-1	0,9963	0,99912	0,99985	0,83223	0,92109	1	1	0,99994	0,69629	0,6798	0,95369
H	-1	0,9963	0,99912	0,99985	0,83223	0,92109	1	1	0,99994	0,69628	0,6798	0,95369
I	-0,99994	0,99625	0,99907	0,99977	0,83216	0,92103	0,99994	0,99994	1	0,69622	0,67974	0,95362
J	-0,69629	0,69525	0,696	0,69622	0,932	0,81469	0,69629	0,69628	0,69622	1	0,99164	0,76129
K	-0,6798	0,67982	0,67974	0,68003	0,91605	0,7996	0,6798	0,6798	0,67974	0,99164	1	0,74593
L	-0,95369	0,95565	0,95474	0,95382	0,90328	0,98014	0,95369	0,95369	0,95362	0,76129	0,74593	1

Figure 11 – Spearman Correlations – Normal Experimental Variants in VIS Range [400-700nm]

A(X)	B(Y)	C(Y)	D(Y)	E(Y)	F(Y)	G(Y)	H(Y)	I(Y)	J(Y)	K(Y)	L(Y)	M(Y)
	A	B	C	D	E	F	G	H	I	J	K	L
Kendall Correlations	Kendall Correlations	Kendall Correlations	Kendall Correlations	Kendall Correlations	Kendall Correlations	Kendall Correlations	Kendall Correlations	Kendall Correlations	Kendall Correlations	Kendall Correlations	Kendall Correlations	Kendall Correlations
A	1	-0,96556	-0,98744	-0,99442	-0,67274	-0,80842	-0,9999	-0,99979	-0,99756	-0,50619	-0,48587	-0,87341
B	-0,96556	1	0,97825	0,96363	0,67322	0,80243	0,96546	0,96534	0,96332	0,51575	0,50212	0,86815
C	-0,98744	0,97825	1	0,98444	0,67207	0,80763	0,98734	0,98723	0,9852	0,50893	0,49228	0,87249
D	-0,99442	0,96363	0,98444	1	0,67083	0,80602	0,99447	0,99447	0,99282	0,50455	0,48491	0,87124
E	-0,67274	0,67322	0,67207	0,67083	1	0,86422	0,67271	0,67249	0,67014	0,8334	0,80847	0,79906
F	-0,80842	0,80243	0,80763	0,80602	0,86422	1	0,8084	0,80819	0,80589	0,69782	0,6755	0,92145
G	-0,9999	0,96546	0,98734	0,99447	0,67271	0,8084	1	0,99973	0,99755	0,50615	0,48583	0,87339
H	-0,99979	0,96534	0,98723	0,99447	0,67249	0,80819	0,99973	1	0,9976	0,50594	0,4856	0,87318
I	-0,99756	0,96332	0,9852	0,99282	0,67014	0,80589	0,99755	0,9976	1	0,50373	0,4834	0,87095
J	-0,50619	0,51575	0,50893	0,50455	0,8334	0,69782	0,50615	0,50594	0,50373	1	0,95099	0,63297
K	-0,48587	0,50212	0,49228	0,48491	0,80847	0,6755	0,48583	0,4856	0,4834	0,95099	1	0,61169
L	-0,87341	0,86815	0,87249	0,87124	0,79906	0,92145	0,87339	0,87318	0,87095	0,63297	0,61169	1

Figure 12 – Kendall Correlations – Normal Experimental Variants in VIS Range [400-700nm]

Statistical calculation elements for activated variants V_0A-$V_{10}A$ (based on molecular absorbance data in the UV range, 190 nm – 400 nm)

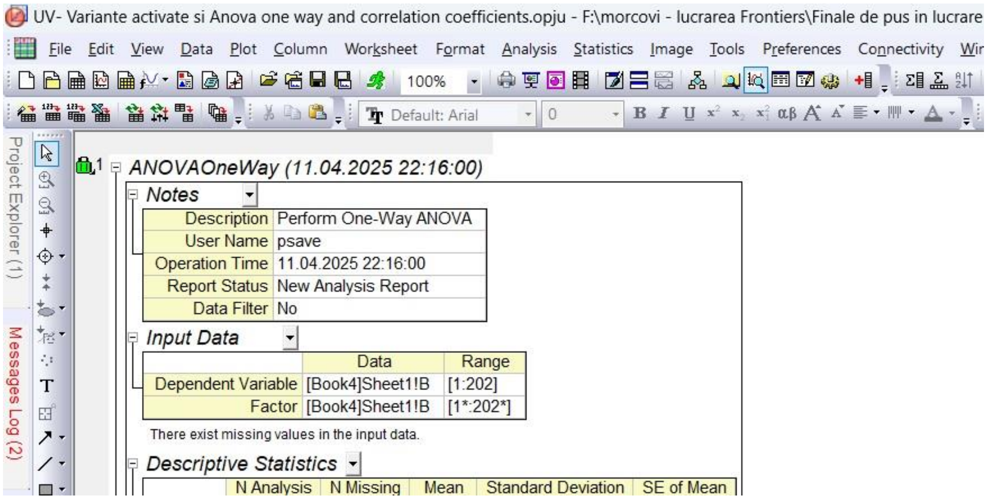

Figure 13 – Input Data for ANOVA One Way – Activated Experimental Variants in UV Range [190-400nm]

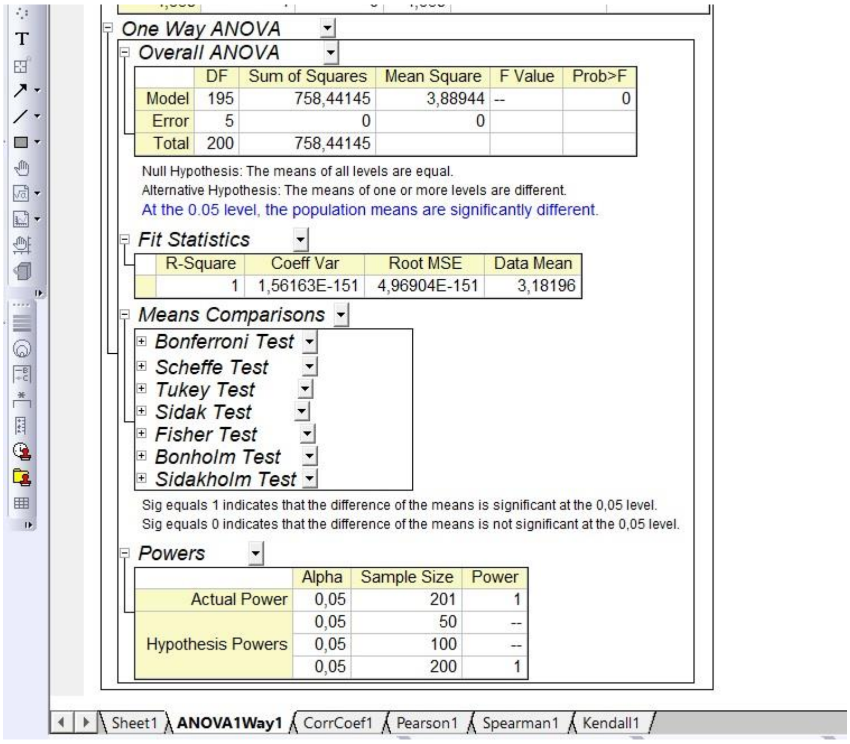

One Way ANOVA

Overall ANOVA

	DF	Sum of Squares	Mean Square	F Value	Prob>F
Model	195	758,44145	3,88944	--	0
Error	5	0	0		
Total	200	758,44145			

Null Hypothesis: The means of all levels are equal.
Alternative Hypothesis: The means of one or more levels are different.
At the 0.05 level, the population means are significantly different.

Fit Statistics

R-Square	Coeff Var	Root MSE	Data Mean
1	1,56163E-151	4,96904E-151	3,18196

Means Comparisons

- Bonferroni Test
- Scheffe Test
- Tukey Test
- Sidak Test
- Fisher Test
- Bonholm Test
- Sidakholm Test

Sig equals 1 indicates that the difference of the means is significant at the 0,05 level.
Sig equals 0 indicates that the difference of the means is not significant at the 0,05 level.

Powers

	Alpha	Sample Size	Power
Actual Power	0,05	201	1
Hypothesis Powers	0,05	50	--
	0,05	100	--
	0,05	200	1

Figure 14 – Overall One Way ANOVA– Activated Experimental Variants in UV Range [190-400nm]

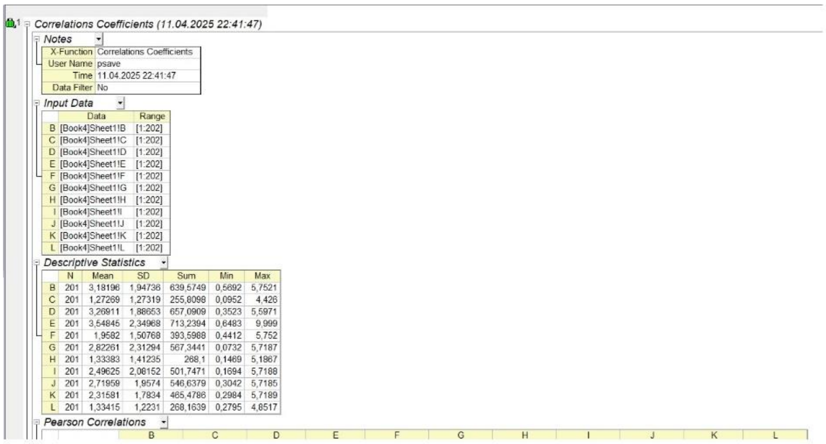

Correlations Coefficients (11.04.2025 22:41:47)

Notes

X-Function	Correlations Coefficients
User Name	psave
Time	11.04.2025 22:41:47
Data Filter	No

Input Data

	Data	Range
B	[Book4]Sheet1!B	[1:202]
C	[Book4]Sheet1!C	[1:202]
D	[Book4]Sheet1!D	[1:202]
E	[Book4]Sheet1!E	[1:202]
F	[Book4]Sheet1!F	[1:202]
G	[Book4]Sheet1!G	[1:202]
H	[Book4]Sheet1!H	[1:202]
I	[Book4]Sheet1!I	[1:202]
J	[Book4]Sheet1!J	[1:202]
K	[Book4]Sheet1!K	[1:202]
L	[Book4]Sheet1!L	[1:202]

Descriptive Statistics

	N	Mean	SD	Sum	Min	Max
B	201	3,18196	1,94736	639,5749	0,5692	5,7521
C	201	1,27269	1,27319	255,8098	0,0952	4,426
D	201	3,26911	1,88653	657,0909	0,3523	5,5971
E	201	3,54845	2,34968	713,2394	0,6483	9,999
F	201	1,9582	1,50768	393,5988	0,4412	5,752
G	201	2,82261	2,31294	567,3441	0,0732	5,7187
H	201	1,33383	1,41235	268,1	0,1469	5,1867
I	201	2,49625	2,08152	501,7471	0,1694	5,7188
J	201	2,71959	1,9574	546,6379	0,3042	5,7185
K	201	2,31581	1,7834	465,4786	0,2984	5,7189
L	201	1,33415	1,2231	268,1639	0,2795	4,8517

Pearson Correlations

	B	C	D	E	F	G	H	I	J	K	L

Figure 15 – Correlations Coefficients– Activated Experimental Variants in UV Range [190-400nm]

A(X)	B(Y)	C(Y)	D(Y)	E(Y)	F(Y)	G(Y)	H(Y)	I(Y)	J(Y)	K(Y)	L(Y)
	B	C	D	E	F	G	H	I	J	K	L
Pearson Correlations	Pearson Correlations	Pearson Correlations	Pearson Correlations	Pearson Correlations	Pearson Correlations	Pearson Correlations	Pearson Correlations	Pearson Correlations	Pearson Correlations	Pearson Correlations	Pearson Correlations
B	1	0,7109	0,87242	0,9273	0,77341	0,99202	0,6976	0,93791	0,97763	0,92278	0,69057
C	0,7109	1	0,60083	0,59371	0,97529	0,69971	0,97587	0,69327	0,74302	0,84115	0,97101
D	0,87242	0,60083	1	0,83594	0,61812	0,83278	0,53233	0,76587	0,80511	0,73622	0,53381
E	0,9273	0,59371	0,83594	1	0,651	0,91938	0,5769	0,85994	0,90094	0,82291	0,57339
F	0,77341	0,97529	0,61812	0,651	1	0,76189	0,9642	0,71538	0,79107	0,90309	0,95674
G	0,99202	0,69971	0,83278	0,91938	0,76189	1	0,68912	0,95014	0,98077	0,91088	0,68315
H	0,6976	0,97587	0,53233	0,5769	0,9642	0,68912	1	0,68142	0,73456	0,87191	0,99045
I	0,93791	0,69327	0,76587	0,85994	0,71538	0,95014	0,68142	1	0,97506	0,86369	0,66775
J	0,97763	0,74302	0,80511	0,90094	0,79107	0,98077	0,73456	0,97506	1	0,92842	0,72326
K	0,92278	0,84115	0,73622	0,82291	0,90309	0,91088	0,87191	0,86369	0,92842	1	0,8539
L	0,69057	0,97101	0,53381	0,57339	0,95674	0,68315	0,99045	0,66775	0,72326	0,8539	1

Figure 16– Pearson Correlations – Activated Experimental Variants in UV Range [190-400nm]

A(X)	B(Y)	C(Y)	D(Y)	E(Y)	F(Y)	G(Y)	H(Y)	I(Y)	J(Y)	K(Y)	L(Y)
	B	C	D	E	F	G	H	I	J	K	L
Spearman Correlations	Spearman Correlations	Spearman Correlations	Spearman Correlations	Spearman Correlations	Spearman Correlations	Spearman Correlations	Spearman Correlations	Spearman Correlations	Spearman Correlations	Spearman Correlations	Spearman Correlations
B	1	0,84412	0,85362	0,96245	0,90456	0,97528	0,8987	0,90136	0,93879	0,91729	0,89371
C	0,84412	1	0,7546	0,82423	0,97112	0,83178	0,97171	0,81154	0,85026	0,91701	0,9708
D	0,85362	0,7546	1	0,85422	0,75053	0,87122	0,74526	0,73496	0,74499	0,72121	0,73983
E	0,96245	0,82423	0,85422	1	0,88171	0,96578	0,87828	0,90019	0,93146	0,89785	0,8749
F	0,90456	0,97112	0,75053	0,88171	1	0,87801	0,99911	0,84721	0,9047	0,97237	0,99812
G	0,97528	0,83178	0,87122	0,96578	0,87801	1	0,87299	0,91843	0,94295	0,8914	0,86807
H	0,8987	0,97171	0,74526	0,87828	0,99911	0,87299	1	0,84414	0,90179	0,97144	0,99928
I	0,90136	0,81154	0,73496	0,90019	0,84721	0,91843	0,84414	1	0,97175	0,89809	0,84122
J	0,93879	0,85026	0,74499	0,93146	0,9047	0,94295	0,90179	0,97175	1	0,9384	0,89904
K	0,91729	0,91701	0,72121	0,89785	0,97237	0,8914	0,97144	0,89809	0,9384	1	0,97013
L	0,89371	0,9708	0,73983	0,8749	0,99812	0,86807	0,99928	0,84122	0,89904	0,97013	1

Figure 17– Spearman Correlations – Activated Experimental Variants in UV Range [190-400nm]

A(X)	B(Y)	C(Y)	D(Y)	E(Y)	F(Y)	G(Y)	H(Y)	I(Y)	J(Y)	K(Y)	L(Y)
	B	C	D	E	F	G	H	I	J	K	L
Kendall Correlations	Kendall Correlations	Kendall Correlations	Kendall Correlations	Kendall Correlations	Kendall Correlations	Kendall Correlations	Kendall Correlations	Kendall Correlations	Kendall Correlations	Kendall Correlations	Kendall Correlations
B	1	0,72134	0,73371	0,87423	0,80118	0,92463	0,79402	0,79224	0,85104	0,81551	0,78765
C	0,72134	1	0,62961	0,68154	0,89659	0,70471	0,90236	0,68673	0,73738	0,80648	0,89619
D	0,73371	0,62961	1	0,71185	0,65552	0,74446	0,64995	0,61178	0,63214	0,61963	0,64378
E	0,87423	0,68154	0,71185	1	0,74979	0,87914	0,746	0,77479	0,82169	0,77253	0,74063
F	0,80118	0,89659	0,65552	0,74979	1	0,76565	0,99025	0,73165	0,80239	0,90064	0,98507
G	0,92463	0,70471	0,74446	0,87914	0,76565	1	0,75968	0,81721	0,8564	0,77952	0,75331
H	0,79402	0,90236	0,64995	0,746	0,99025	0,75968	1	0,72607	0,79642	0,89527	0,99264
I	0,79224	0,68673	0,61178	0,77479	0,73165	0,81721	0,72607	1	0,90231	0,80513	0,7199
J	0,85104	0,73738	0,63214	0,82169	0,80239	0,8564	0,79642	0,90231	1	0,85338	0,79085
K	0,81551	0,80648	0,61963	0,77253	0,90064	0,77952	0,89527	0,80513	0,85338	1	0,89009
L	0,78765	0,89619	0,64378	0,74063	0,98507	0,75331	0,99264	0,7199	0,79085	0,89009	1

Figure 18 – Kendall Correlations – Activated Experimental Variants in UV Range [190-400nm]

Statistical calculation elements for activated variants V_0A-$V_{10}A$ (based on molecular absorbance data in the Visible range, 400 nm-700 nm)

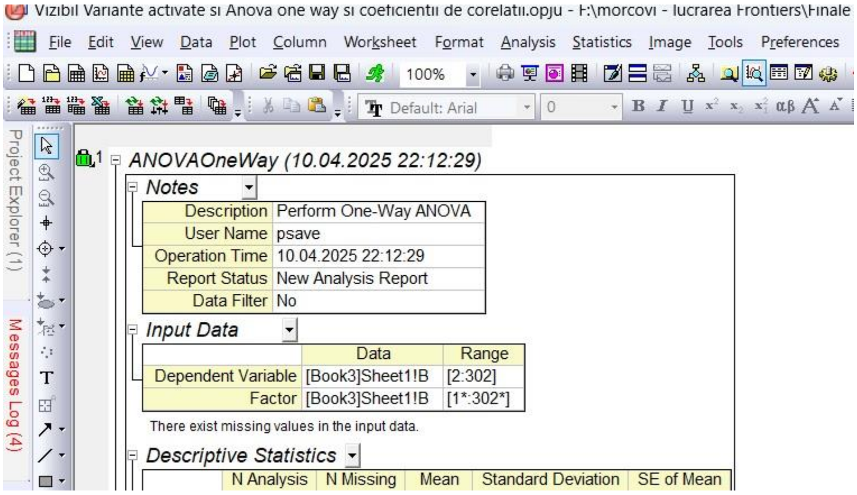

ANOVAOneWay (10.04.2025 22:12:29)

Notes

Description	Perform One-Way ANOVA
User Name	psave
Operation Time	10.04.2025 22:12:29
Report Status	New Analysis Report
Data Filter	No

Input Data

	Data	Range
Dependent Variable	[Book3]Sheet1!B	[2:302]
Factor	[Book3]Sheet1!B	[1*:302*]

There exist missing values in the input data.

Descriptive Statistics

	N Analysis	N Missing	Mean	Standard Deviation	SE of Mean

Figure 19 – Input Data for ANOVA One Way – Activated Experimental Variants in VIS Range [400-700nm]

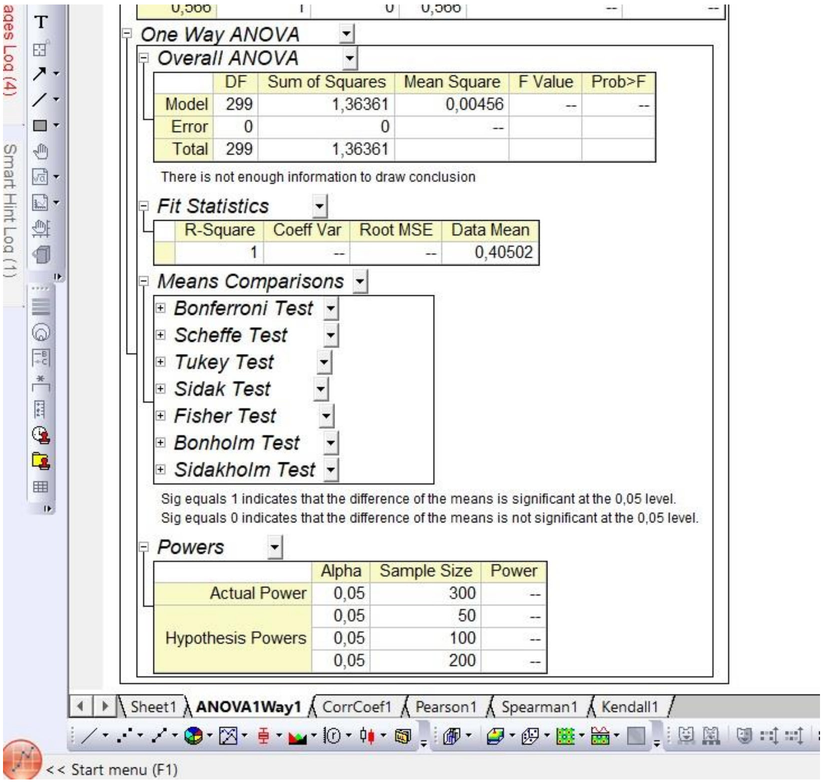

One Way ANOVA

Overall ANOVA

	DF	Sum of Squares	Mean Square	F Value	Prob>F
Model	299	1,36361	0,00456	--	--
Error	0	0	--		
Total	299	1,36361			

There is not enough information to draw conclusion

Fit Statistics

R-Square	Coeff Var	Root MSE	Data Mean
1	--	--	0,40502

Means Comparisons

- Bonferroni Test
- Scheffe Test
- Tukey Test
- Sidak Test
- Fisher Test
- Bonholm Test
- Sidakholm Test

Sig equals 1 indicates that the difference of the means is significant at the 0,05 level.
Sig equals 0 indicates that the difference of the means is not significant at the 0,05 level.

Powers

	Alpha	Sample Size	Power
Actual Power	0,05	300	--
Hypothesis Powers	0,05	50	--
	0,05	100	--
	0,05	200	--

Figure 20 – Overall One Way ANOVA– Activated Experimental Variants in VIS Range [400-700nm]

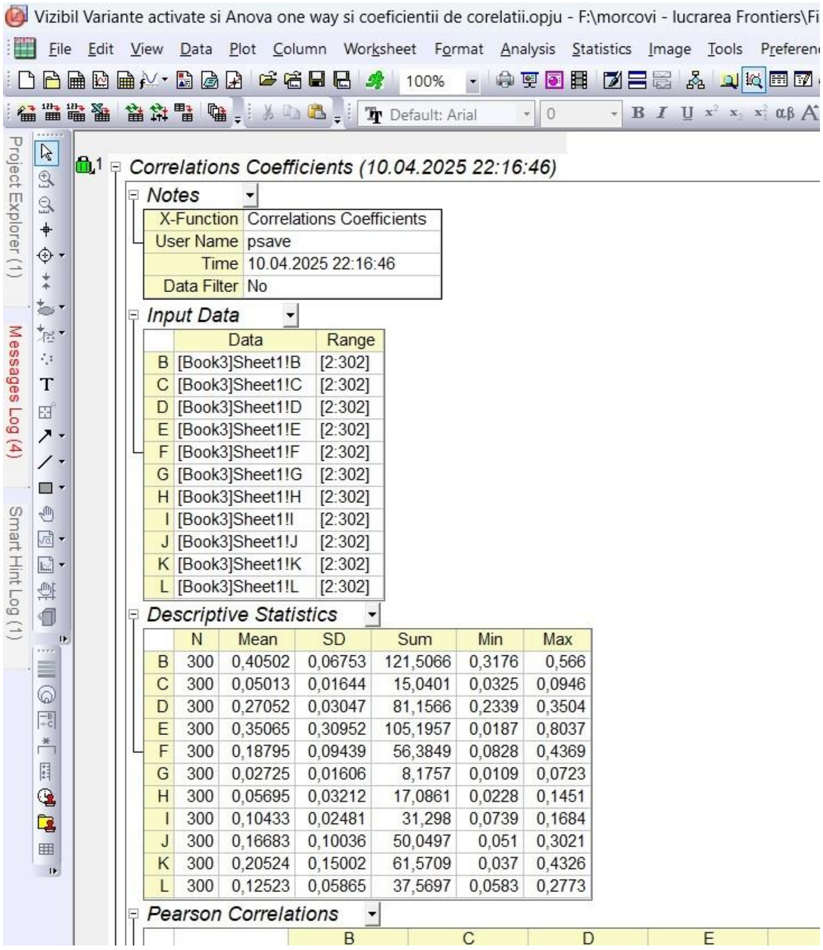

	Data	Range
B	[Book3]Sheet1!B	[2:302]
C	[Book3]Sheet1!C	[2:302]
D	[Book3]Sheet1!D	[2:302]
E	[Book3]Sheet1!E	[2:302]
F	[Book3]Sheet1!F	[2:302]
G	[Book3]Sheet1!G	[2:302]
H	[Book3]Sheet1!H	[2:302]
I	[Book3]Sheet1!I	[2:302]
J	[Book3]Sheet1!J	[2:302]
K	[Book3]Sheet1!K	[2:302]
L	[Book3]Sheet1!L	[2:302]

	N	Mean	SD	Sum	Min	Max
B	300	0,40502	0,06753	121,5066	0,3176	0,566
C	300	0,05013	0,01644	15,0401	0,0325	0,0946
D	300	0,27052	0,03047	81,1566	0,2339	0,3504
E	300	0,35065	0,30952	105,1957	0,0187	0,8037
F	300	0,18795	0,09439	56,3849	0,0828	0,4369
G	300	0,02725	0,01606	8,1757	0,0109	0,0723
H	300	0,05695	0,03212	17,0861	0,0228	0,1451
I	300	0,10433	0,02481	31,298	0,0739	0,1684
J	300	0,16683	0,10036	50,0497	0,051	0,3021
K	300	0,20524	0,15002	61,5709	0,037	0,4326
L	300	0,12523	0,05865	37,5697	0,0583	0,2773

	B	C	D	E

Figure 21– Correlations Coefficients– Activated Experimental Variants in VIS Range [400-700nm]

A(X)	B(Y)	C(Y)	D(Y)	E(Y)	F(Y)	G(Y)	H(Y)	I(Y)	J(Y)	K(Y)	L(Y)
	B	C	D	E	F	G	H	I	J	K	L
Pearson Correlations	Pearson Correlations	Pearson Correlations	Pearson Correlations	Pearson Correlations	Pearson Correlations	Pearson Correlations	Pearson Correlations	Pearson Correlations	Pearson Correlations	Pearson Correlations	Pearson Correlations
B	1	0,99032	0,99849	0,93587	0,99331	0,98882	0,9887	0,99781	0,91905	0,82623	0,99481
C	0,99032	1	0,99231	0,89314	0,99932	0,99933	0,99954	0,99684	0,86711	0,75296	0,99892
D	0,99849	0,99231	1	0,9344	0,99396	0,99209	0,99053	0,99755	0,91666	0,82152	0,99498
E	0,93587	0,89314	0,9344	1	0,90052	0,89508	0,88895	0,91479	0,99353	0,96232	0,9046
F	0,99331	0,99932	0,99396	0,90052	1	0,99832	0,99896	0,99847	0,87666	0,76574	0,99988
G	0,98882	0,99933	0,99209	0,89508	0,99832	1	0,9994	0,99532	0,86991	0,75647	0,99774
H	0,9887	0,99954	0,99053	0,88895	0,99896	0,9994	1	0,99569	0,86371	0,74855	0,99841
I	0,99781	0,99684	0,99755	0,91479	0,99847	0,99532	0,99569	1	0,89401	0,79013	0,99911
J	0,91905	0,86711	0,91666	0,99353	0,87666	0,86991	0,86371	0,89401	1	0,98038	0,88145
K	0,82623	0,75296	0,82152	0,96232	0,76574	0,75647	0,74855	0,79013	0,98038	1	0,77247
L	0,99481	0,99892	0,99498	0,9046	0,99988	0,99774	0,99841	0,99911	0,88145	0,77247	1

Figure 22 – Pearson Correlations – Activated Experimental Variants in VIS Range [400-700nm]

A(X)	B(Y)	C(Y)	D(Y)	E(Y)	F(Y)	G(Y)	H(Y)	I(Y)	J(Y)	K(Y)	L(Y)
	B	C	D	E	F	G	H	I	J	K	L
Spearman Correlations	Spearman Correlations	Spearman Correlations	Spearman Correlations	Spearman Correlations	Spearman Correlations	Spearman Correlations	Spearman Correlations	Spearman Correlations	Spearman Correlations	Spearman Correlations	Spearman Correlations
B	1	0,99964	0,99991	0,98231	0,99998	0,99926	0,99986	0,99998	0,9454	0,87748	0,99998
C	0,99964	1	0,99954	0,98198	0,99964	0,99911	0,99947	0,99966	0,94503	0,87711	0,99965
D	0,99991	0,99954	1	0,98246	0,99985	0,99927	0,99977	0,9999	0,9452	0,87761	0,99985
E	0,98231	0,98198	0,98246	1	0,98229	0,98161	0,98217	0,9823	0,94502	0,89952	0,98229
F	0,99998	0,99964	0,99985	0,98229	1	0,99924	0,99988	0,99997	0,94539	0,87746	0,99999
G	0,99926	0,99911	0,99927	0,98161	0,99924	1	0,99943	0,99928	0,94472	0,8768	0,99924
H	0,99986	0,99947	0,99977	0,98217	0,99988	0,99943	1	0,99985	0,94532	0,87736	0,99987
I	0,99998	0,99966	0,9999	0,9823	0,99997	0,99928	0,99985	1	0,94538	0,87746	0,99997
J	0,9454	0,94503	0,9452	0,94502	0,94539	0,94472	0,94532	0,94538	1	0,95251	0,94539
K	0,87748	0,87711	0,87761	0,89952	0,87746	0,8768	0,87736	0,87746	0,95251	1	0,87746
L	0,99998	0,99965	0,99985	0,98229	0,99999	0,99924	0,99987	0,99997	0,94539	0,87746	1

Figure 23 – Spearman Correlations – Activated Experimental Variants in UV Range [400-700nm]

A(X)	B(Y)	C(Y)	D(Y)	E(Y)	F(Y)	G(Y)	H(Y)	I(Y)	J(Y)	K(Y)	L(Y)
	B	C	D	E	F	G	H	I	J	K	L
Kendall Correlations	Kendall Correlations	Kendall Correlations	Kendall Correlations	Kendall Correlations	Kendall Correlations	Kendall Correlations	Kendall Correlations	Kendall Correlations	Kendall Correlations	Kendall Correlations	Kendall Correlations
B	1	0,99154	0,99736	0,91011	0,99946	0,98773	0,99669	0,99881	0,85823	0,72705	0,99911
C	0,99154	1	0,99009	0,90331	0,9916	0,98636	0,99	0,99181	0,85036	0,71918	0,99162
D	0,99736	0,99009	1	0,91065	0,99689	0,98766	0,99529	0,99703	0,85576	0,72762	0,99673
E	0,91011	0,90331	0,91065	1	0,90968	0,89937	0,90719	0,9094	0,85482	0,81372	0,90963
F	0,99946	0,9916	0,99689	0,90968	1	0,98726	0,99709	0,99847	0,85818	0,7266	0,99962
G	0,98773	0,98636	0,98766	0,89937	0,98726	1	0,98978	0,98831	0,84755	0,71619	0,98734
H	0,99669	0,99	0,99529	0,90719	0,99709	0,98978	1	0,99627	0,8565	0,72433	0,99695
I	0,99881	0,99181	0,99703	0,9094	0,99847	0,98831	0,99627	1	0,85725	0,72628	0,99845
J	0,85823	0,85036	0,85576	0,85482	0,85818	0,84755	0,8565	0,85725	1	0,8679	0,85812
K	0,72705	0,71918	0,72762	0,81372	0,7266	0,71619	0,72433	0,72628	0,8679	1	0,72644
L	0,99911	0,99162	0,99673	0,90963	0,99962	0,98734	0,99695	0,99845	0,85812	0,72644	1

Figure 24 – Kendall Correlations – Activated Experimental Variants in VIS Range [400-700nm]

For each series of experimental variants (normal and activated), for each measurement domain (UV, respectively VIS), a data analysis table, a OneWay ANOVA analysis table, a table with correlation coefficients and one for each correlation (Pearson, Spearman, Kendall) were constructed. These tables are presented in Figures 1-24 of this file.

Analysis of variance (ANOVA) is a statistical method used to compare means between two or more groups. One-way ANOVA is a technique commonly used to analyse the variance of a single continuous variable between two or more categorical groups. This statistical method is used to test hypotheses and draw conclusions about differences between groups.

The technique was used to divide the total variability of the data into two components: the variation between groups (due to the activation treatment) and the variation within each group (due to random variation and individual differences from V0 or V0A).

Anova tests work best when the data are normally distributed and the variances of the groups are equal. If these assumptions are not met, alternative nonparametric tests can be used instead.

Generally, within the same group, the size of the effect produced by additivity was measured using eta-squared (η^2), which represents the proportion of the total variation in the dependent variable that is accounted for by differences between groups.

Using mainly natural additives, this indicator was of medium and small effect.

Small effect: $\eta^2 < 0.01$

Medium effect: $0.01 \leq \eta^2 < 0.06$

The Null Hypothesis: the means of all levels were equal.

Alternative Hypothesis: the means of one or more levels were different. At the 0.05 level, the population means were significantly different.

The correlation coefficient is a key tool in correlation analysis. It is a numerical value that ranges from -1 to +1, indicating both the strength and direction of the relationship between two variables. The most commonly used correlation coefficient is the Pearson correlation, which is ideal for continuous, linear relationships between variables. There was only one perfect positive correlation (+1) that indicated that both variables increased in the same way together (V5 and V6). No perfect negative correlations (-1) were recorded.

Very interesting is the appearance of a maximum value correlation in the Spearman type recorded for the visible range in the variants that used natural sweeteners of the sugar type (V5) and, respectively, stevioside (V6).

Other correlation coefficients include Spearman's Rank Correlation (used for ordinal or nonlinear data) and Kendall's Tau (used for classifying data with fewer assumptions about the data distribution).

Strongly different effects are recorded, in certain measurement ranges (UV), in the action of salicylic acid (V2) and stevioside (V6). These additives should not be used together, in the same variant, manifesting antagonistic mechanisms and post-use effects.

In relation to the Pearson correlations, high values were recorded in experimental variants that used acetic acid as a preservative (V1), beta-carotene – as a dye (V4), white sugar (V5) and low values when adding carmine red and stevioside (V9), carmine red (V3).

From the data analysis, the following were recorded:

Strong positive correlations (+0.7 to +1.0): As one variable increases, the other also increases significantly.

Strong negative correlations (-0.7 to -1.0): As one variable increases, the other decreases significantly.

The carmine red dye can induce large changes in the control variant V0.

The activated variants were characterized by positive correlations, higher values of the partial correlation coefficients and proved an increased stability during storage.

In summary, as can be seen from the statistical results, negative correlations appear at the basic variant V0, which are transformed - into positive correlations (as they appear at V0A, after activation).

Statistical tools are important and can be of great use when we want to find the best recipe variants (especially for personalized nutrition, for consumers with certain conditions). In a future paper, these changes will be documented, also highlighting the differences recorded by changing the order in which the additives are introduced and how activation (in plasma, magnetic and gravitational fields) influences the bromatology of functional foods.

Differences (±) recorded from the Witness Variants in the statistical factor of determination R-squared for normal (V0-V10) and activated (V0A-V10A) experimental variants in the UV-Vis (190-700nm) ranges

Table 3-Supplementary Material

Experimental Variants	R^2 Value	Differences (±) recorded from the Witness Variants	Experimental Variants	R^2 Value	Differences (±) recorded from the Witness Variants
V_0	0.9398	0.0000	V_{0A}	0.9710	0.0000
V_1	0.9143	**-0.0255**	V_{1A}	0.9655	**-0.0055**
V_2	0.8893	-0.0505	V_{2A}	0.9343	-0.0367
V_3	0.8073	**-0.1325**	V_{3A}	0.8983	**-0.0727**
V_4	0.9140	**-0.0258**	V_{4A}	0.9830	+0.0120
V_5	0.8723	-0.0675	V_{5A}	0.9465	-0.0245
V_6	0.8716	-0.0682	V_{6A}	0.9742	**+0.0032**
V_7	0.9035	-0.0363	V_{7A}	0.8469	**-0.1241**
V_8	0.8165	**-0.1233**	V_{8A}	0.9304	**-0.0406**
V_9	0.7049	**-0.2349**	V_{9A}	0.9636	**-0.0074**
V_{10}	0.9083	**-0.0315**	V_{10A}	0.9897	+0.0187

- **In green font = the smallest differences from Control Variant V_0 or V_{0A}**
- **In red font = the biggest differences from the Control Variant V_0 or V_{0A}**

As demonstrated in Table 1 (Supplementary Material), the statistical analysis of the influences recorded by food additives on the basic chemical composition of carrot juice results.

It is evident that stability effects are recorded in comparison to the control variant V_0 in the variants that have been added with acetic acid (a preservative in variant V_1), β-carotene (the dye used in V_4) and the additives present in the commercial version of organic juice (V_{10}).

A stability effect is also recorded when salicylic acid is used as a preservative (V_2).

Antagonistic effects (with large changes in the concentrations of the basic compounds) are recorded when using the red-carmine additive, in combination with steviosides (in variant V_9), with sugar (in variant V_8) or alone (in variant V_3).

The innovative plasma field activation technology employed in this study has been shown to induce compositional changes that are significantly more subtle than those observed in the control variant V_{0A}. It is evident that the variants incorporating stevioside (V_{6A}), acetic acid (V_{1A}), and carmine red in conjunction with stevioside (V_{8A}) exhibit a high degree of stability.

A multitude of alterations also ensue as a consequence of antagonistic effects, when the utilisation of synthetic fructose (V_{7A}) in combination with carmine red (V_{3A}) is employed. The present study also recorded additional activation effects when stevioside (V_{6A}) and β-carotene (V_{4A}) were used, the latter being a constituent of the recipe of the commercial organic juice (V_{10A}).

The comparative analysis of the data obtained at V_0, V_1 and V_2 was utilised to ascertain the influence of preservatives in carrot juice. It was determined that the optimal variant was V_1, which corresponded to juice preserved with 1% acetic acid.

The present study was conducted with the objective of determining the influence of dyes on carrot juice. To this end, a comparative study was conducted on the values V_0; V_3 and V_4. The results of this study indicated that the best variant was V4, in which β-carotene was used.

A thorough analysis of the data obtained for variants V_0; V_5; V_6 and V_7 was conducted, with the objective of identifying the most effective sweetening variant. The results of this analysis indicated that variant V_5 (the carrot juice variant sweetened with sugar) was the most efficacious.

In the context of field processing, the innovative technology was found to be most effective when utilising the additive variants V_{1A} (acetic acid at a concentration of 1%) and V_{4A} (β-carotene). However, when it came to sweetening, stevioside (from V_{6A}) emerged as the optimal choice.

In the present study, the effect of various sweetening agents on the stability of red-carmine dye in carrot juice from field-activated variants (V_{9A}) was investigated. The results demonstrated that stevioside was the only sweetener capable of stabilising the red-carmine dye in the carrot juice.
