# Supplementary material for: Synergistic effects produced by certain antioxidants in valuable functional foods from the Romanian markets
Source: Front Nutr. 2025 Jun 19;12:1558597. doi: 10.3389/fnut.2025.1558597 (PMC12221894; doi:10.3389/fnut.2025.1558597)
Supplement: Supplementary file 4 [file Data_Sheet_3.pdf]

Influence of the use of innovative technologies and the use of statistics/mathematical modeling in the identification of synergistic and/or antagonistic effects produced by antioxidants in the chemical composition of some Romanian functional foods upon additivation (case study being performed on carrot juice). Summary

Benefits of implementing the proposed new processing technology carrot juice - functional food:

- Development of a higher yield at an improved extraction (SFE -Supercritical Fluid Extraction) of the main antioxidants - valuable bio-compounds from carrot juice. In this case, SFE - Supercritical Fluid Extraction, with supercritical agent -CO2, in a $_{CO2}$ plasma field was used;
- Additional activation of bio-compounds in the plasma field by generators and membranes;
- Stabilization of final products (carrot juice with high nutrient density - functional food) - in order to extend the shelf life and storage life of juices;
- Stabilization - over a longer period of time - of the specific redox potential of several redox systems within and at the juice/air interface (by balancing the concentrations of oxidized and reduced forms of NAD- or FMN-dependent oxido-reductase coenzymes);
- Developing innovative recipes - for functional foods (activated carrot juices) - in which valuable compounds (antioxidants, provitamins, vitamins) are absorbed slowly - without major caloric changes - into the body of consumers;
- Improving existing correlations and synergistic effects - occurring when carrot-functional food juices are added;
- The technology can be used as a preventive/corrective method against attacks by potentially pathogenic micro-organisms - for consumer health;
- It can also bring added food safety in the bromatology of the final forms of carrot juices

Advantages of using statistical and/or mathematical modeling tools in the identification of synergistic/antagonistic effects occurring in carrot juice additive - functional food:

- Defining the best, personalized additive options - for consumers with different nutritional problems (by selecting and promoting the best options);

- Analysis of positive or negative correlations - to prevent some antagonistic effects and to promote some synergistic effects - on the transfer of mass, heat, momentum - from technological operations;
- Study of compositional changes and product bromatology that may occur when food additives, ingredients or processing aids are added (by analyzing the molecular absorption spectra curves of the main valuable biocompounds in juices, by monitoring trend lines and R^2 coefficients of statistical determination);
- Accurate choice of personalized prescriptions and their promotion to different types of consumers within national public health policies;
- The realization of mathematical models to transfer (respecting the principles of accuracy, repeatability, reproducibility) the obtained results, to adapt and apply them to other foodstuffs - of the same category;
- Check, at various intervals, for synergistic or antagonistic effects that may occur in the product (during storage or transportation, for example). These effects may have a major influence on the shelf-life and keeping qualities of some products, but also on the absorption of antioxidants, i.e. the rate of conversion of provitamins into vitamins in the consumer's digestive system.
